# The correct way to test the hypothesis that racial categorization is a byproduct of an evolved alliance-tracking capacity

**DOI:** 10.1038/s41598-021-82975-x

**Published:** 2021-02-09

**Authors:** David Pietraszewski

**Affiliations:** Center for Adaptive Rationality, Max Planck Institute for Human Development, Lentzeallee 94, 14195 Berlin, Germany

**Keywords:** Psychology, Human behaviour

## Abstract

The project of identifying the cognitive mechanisms or information-processing functions that cause people to categorize others by their race is one of the longest-standing and socially-impactful scientific issues in all of the behavioral sciences. This paper addresses a critical issue with one of the few hypotheses in this area that has thus far been successful—*the alliance hypothesis of race*—which had predicted a set of experimental circumstances that appeared to selectively target and modify people’s implicit categorization of others by their race. Here, we will show why the evidence put forward in favor of this hypothesis was not in fact evidence in support of the hypothesis, contrary to common understanding. We will then provide the necessary and crucial tests of the hypothesis in the context of conflictual alliances, determining if the predictions of the alliance hypothesis of racial categorization in fact hold up to experimental scrutiny. When adequately tested, we find that indeed categorization by race is selectively reduced when crossed with membership in antagonistic alliances—the very pattern predicted by the alliance hypothesis. This finding provides direct experimental evidence that the human mind treats race as proxy for alliance membership, implying that racial categorization does not reflect attention to physical features per se, but rather to social relationships.

## Introduction

Race occupies a central place in the history of the behavioral and cognitive sciences. Few other real-world phenomena can claim to have sustained the attention of the entire field throughout its varied history. Indeed, views of what race is and how to study it reflect the changing views of what the mind is and how to study it: from cataloguing what people associate with race^1^, to viewing the representation of race as an example of perceptual and semantic shortcutting^2,3^, and—with the advent of the cognitive revolution—as either a heuristic or bias^4^, or as an example of the intrinsically-evaluative nature of human cognition^5^.

A watershed moment for understanding the psychology of race perception and categorization occurred in 2001 with the publication of the paper, “Can race be erased?” by Kurzban and colleagues^6^. This paper was important for two reasons. One, it was the first time that a group of researchers had reported a successful experimental decrease in participants’ categorization of others by their race. While previous studies had found it trivially-easy to reduce racial stereotyping (the application of content associated with a category to a particular person), there was no known manipulation that had any effect on the process of racial categorization itself (that is, on the process of assigning a person to that social category in the first place^7,8^). The discovery of a successful manipulation suggested that a new and fundamental insight about the psychology of racial perception and categorization had been uncovered.

Second, the paper demonstrated the utility of adopting an evolutionary approach. The successful manipulation had been discovered by considering what functions would be likely to exist within the mind, given a history of evolution. In particular, Kurzban et al. reasoned that because racial differences are evolutionarily-novel, racial categorization is not likely to be a dedicated function of the mind’s information-processing. Rather, it is likely to be a byproduct. The hypothesis considered by Kurzban et al. was that perhaps racial categorization is a byproduct of an ability to track alliances—something that would be selected for over evolutionary time. Tracking alliances requires (i) representing the units of coordination, cooperation, and competition that exist in one’s social world, and (ii) assigning individuals to these units, as a way to predict who will be affiliated with whom^9,10^.

Kurzban et al. reasoned that perhaps cognitive systems with these functions might, as a byproduct of their operation, also pick up on physical traits that tend to correlate with such patterns of coordination, cooperation, and competition in one’s local social ecology. If true, then this would mean that people categorize by their race because of exposure to racially-segregated and discriminatory social ecologies^11,12^. Race would then serve as a proxy to alliance membership^6,9^. We will refer to this as *the alliance hypothesis* of race.

Crucially, the alliance hypothesis suggests an experimental manipulation that should in fact modify categorization by race: If one can show that race—which presumably has a high prior of being predictive of how people are likely to interact and get along with one another for participants—is shown to be no longer predictive of who is allied with whom in an ongoing interaction, then its psychological importance should be reduced. Consequently, its use as an implicit basis of categorizing people should be reduced or abandoned, particularly when a new basis of predicting alliances is presented.

The Kurzban et al. paper is widely understood as testing and providing support for this prediction of the alliance hypothesis, utilizing the same paradigm that had previously found invariant categorization by race: *the memory confusion paradigm* (see Box 1). In their study, participants observed an antagonistic interaction between the members of two different basketball teams, each of whom supported one another while threatening and mocking the members of the other team. Such behaviors, by hypothesis, should engage the mind’s alliance-tracking. Participants were therefore predicted to categorize the interactants according to their team membership.

**Box 1:**
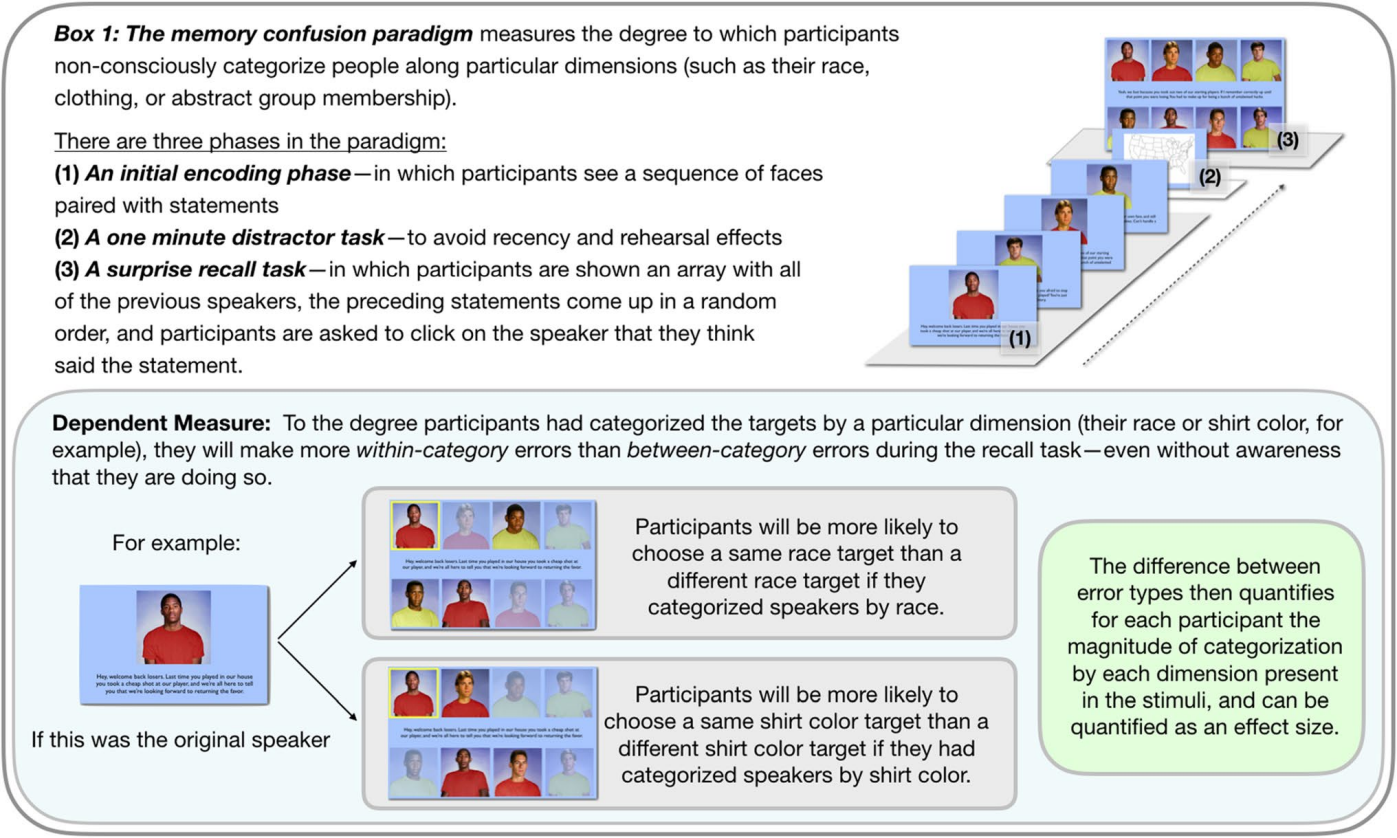
The memory confusion paradigm.

Moreover, and importantly, there were an equal number of Black and White players on each team. If the alliance hypothesis is correct, then this manipulation should reduce categorization by race: If the mind attends to race as a cue to how individuals are likely to interact and get along with one another, then showing that race is not predictive of such relationships should reduce the relative importance of race (at least temporarily). This reduction should then be reflected in lowered levels of categorization.

**Figure 1:**
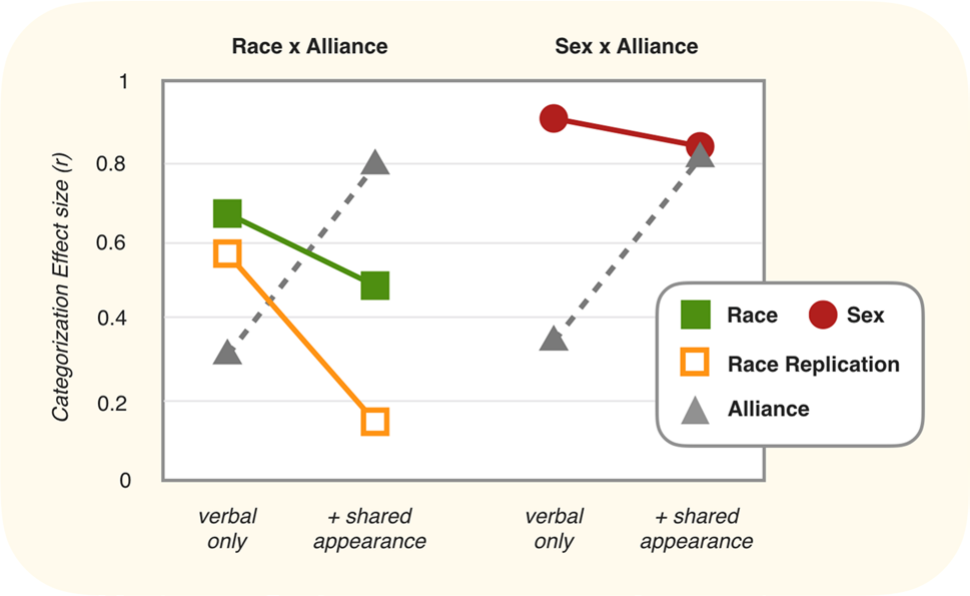
Data reported in Kurzban et al. (2001). *Categorization effect size* describes the degree to which participants within each condition made more within-category memory errors than between-category memory errors. The effect size is calculated as the difference in means weighted by the variance within those means, and is standardized to range between 0 and 1.

In fact, such a reduction in categorization by race was found in Kurzban et al.—specifically, between two different between-subjects manipulations: one in which team membership was not marked by team jersey color (*verbal only*), and a second condition in which jersey color was added to mark team membership (+ *shared appearance*). The left side of Fig. 1 depicts (1) an increase in team categorization and (2) a decrease racial categorization across these two conditions (the y-axis describes the magnitude of categorization as determined by patterns of errors in the memory confusion paradigm):

The reduction in racial categorization depicted on the left side of Fig. 1 seems to be consistent with the alliance hypothesis of race. However, a counterhypothesis remains: Perhaps the reduction in race reflects an attentional shift, such that any non-relevant category would also be reduced under the same circumstances. Such a counterhypothesis would predict that age and sex (which are not hypothesized to be intrinsically alliance cues) will also be reduced if place in the same experimental context. The alliance hypothesis, in contrast, predicts that racial categorization will be *differentially* reduced compared to these other categories. In particular, there are good reasons to suspect that there are dedicated procedures in the mind for attending to both age and sex, as both would have been enduring features of conspecifics within ancestral environments, unlike race. The right side of Fig. 1 depicts Kurzban et al.’s test against this counterhypothesis. In this set of conditions, the same between-subject manipulation that had been applied to race was now also applied to sex. As shown, categorization by sex was slightly lowered, but not quite as much as the reduction in race, consistent with the alliance hypothesis.

This Kurzban et al. study is widely-regarded both within and outside of psychology as demonstrating (1) that the human mind spontaneously keeps track of the coalitional alliances of others (that is, it keeps track of who is on whose side in a conflict), and (2) that racial categorization is a byproduct of this coalition-tracking ability^13–17^. Indeed, it is one of the most frequently-cited empirical findings within Evolutionary Psychology and about the psychology of race in general (cited over 1000 times as of the writing of this paper).

Subsequent studies have been conducted to test the *alliance hypothesis*—for example, in the context of cooperation, rather than antagonism^17^, and also with political affiliations^18^—but these have been largely seen by the scientific community as secondary, follow-up studies. The original Kurzban et al. study is still considered by many to be the primary source of evidence for the hypothesis. It is continually and universally-cited as providing evidence in support of the hypothesis^19,20^—such that any issues with the Kurzban paper are seen as grounds for dismissing the alliance hypothesis in general^21^. As the Kurzban et al. paper goes, so goes the evidence for the alliance hypothesis itself.

Our first goal here is to explain why, in the fullness of time, we now know this to not be true. Although these results of Kurzban et al. seem promising, there is a problem with this study design—a problem that has not been noticed in the over one thousand times that the paper has been cited, nor in replication attempts^22^, and thus has not yet been adequately addressed^17,18^. The problem is that this study design is not even in principle capable of testing the primary predictions of the alliance hypothesis.

In particular, there are two primary predictions of the alliance hypothesis:

1. Categorization by a now-relevant alliance membership (e.g., team membership) will occur.
2. Racial categorization will be differentially lowered when it is shown to be no longer predictive of alliance membership (i.e., when it is crossed with team membership, such that race no longer predicts who is on who’s side).

The problem with the Kurzban et al. study design is that in order to test the second prediction, one would need to know (1) what level of racial categorization one finds in the absence of any alliance information, and then (2) compare that to the level of racial categorization that one finds when race is crossed with team membership. The Kurzban et al. paper is frequently misunderstood as doing this very thing, but this *not* what was done.

Instead, what was compared across conditions was the effect *adding a visual marker of team membership*—not of adding team membership itself. In other words, (and as can be seen in Fig. 1 above) in both between-subjects conditions (that is, the *verbal only* and + *shared appearance* conditions) race was always crossed with team membership. In both conditions the team membership of each player was always clearly discernible based on what each player said and the order in which their photo appeared, as the conversation always alternated back-and-forth between members of the two different teams. This means that in both conditions, it was always clear to participants that race was crossed with team membership.

Consequently, there was never any manipulation of whether or not race was crossed with team membership; this was held constant, rather than varying across conditions. Yet it is exactly this manipulation—comparing race on its own to when race is crossed with team membership—that is required to test the primary prediction that race will be lowered when crossed with alliance membership. It is also this manipulation that one must look at when testing whether race will be lowered *more so* than will other social categories, such as sex or age. Yet no such comparison was ever done in Kurzban et al. Therefore, there is no way to evaluate what the impact of crossing race with alliance membership is—even though this is the primary prediction of the alliance hypothesis.

Moreover, the evidence for the *differential* reduction in race—compared in this case to categorization by sex—was weak in the findings of Kurzban et al. Importantly, one cannot make much of the fact that the absolute levels of categorization by sex are higher than are absolute levels of categorization by race in the data. What we know from past studies is that categorization by sex is typically higher than is categorization by race^23–26^. Furthermore, we know that the absolute level of categorization within these kinds of categorization studies is a function of the particulars of the study implementation details (roughly, in some studies one finds that the effect sizes for important social categories are large, others medium, but in all cases the ordinal relationship between, for example, sex versus race remains). In other words, one should not look at the Kurzban data with an implicit view that the levels of categorization by race and sex would have been equally-high if only the experimental contexts had not been coalitional. But unfortunately, this is how the data are often interpreted.

What this all means is that the informative result in Kurzban et al. is the *change* in categorization across the two experimental conditions, not the absolute value of categorization. And it is here that we find the mixed results: Yes, descriptively categorization by race was lowered more than was sex. But was there a strong *differential* reduction in race compared to sex? Here, different researchers looking at these same data may reasonably arrive at different conclusions. On balance, the data are certainly suggestive of a differential effect on race, but not particularly conclusive. However, even if the data were conclusive, this would still only be a differential effect of race as a consequence of shirt color, not team membership. We are then back to the more fundamental issue that even if we do interpret the reduction in race as stronger than the reduction in sex, we are still looking at a reduction caused by something other than what the alliance hypothesis predicts. Namely, shirt color as opposed to team membership.

The upshot of all of this is that what we can conclude, based on the Kurzban et al. data, is that racial categorization is reduced. But we cannot make any inferences that this was due to race being crossed with alliance membership. And we certainly cannot make any inferences based on these data that racial categorization is a consequence of alliance categorization. (Moreover, it has also come to light that an error correction used in prior memory confusion paradigm studies was faulty, leading to biased effect size estimates^27,28^. The data reported in Kurzban et al. are also subject to this problem. Consequently, we cannot even be confident in the results reported therein.)

In sum, then, the most-cited source of evidence in support of the alliance hypothesis of race—that an antagonistic sports team context engages alliance-tracking and causes a reduction in categorization by race—is in fact not an adequate test of the hypothesis. This leaves an uncomfortable gap in our knowledge: If an antagonistic context does *not* in fact produce the pattern of results predicted by the alliance hypothesis, then the hypothesis would be put into jeopardy. Although there has been subsequent work adequately testing the hypothesis in the context of cooperation and coordination^17,18^, a non-result in the context of antagonism when adequately tested would militate against the hypothesis, and would suggest that these other results may be due to more domain-general effects^18^.

Consequently, we ran and report here a study which provides the first adequate test of the alliance hypothesis in the context of conflict. Critically, the study here (1) establishes a baseline level of categorization by race when is it not yet crossed with any team (alliance) membership, and then (2) compares that level of categorization by race to a condition in which race is now crossed with team membership. This comparison then serves as the critical measure of what happens to racial categorization as a consequence of showing that race is no longer predictive of alliance membership.

In order to test the prediction that racial categorization will be *more* affected by such a manipulation than will other categories, the same comparison was also done with sex. Categorization by sex was measured at a baseline level and then again when sex was crossed with team membership. If the alliance hypothesis of racial categorization is correct, any reduction in categorization by sex across these conditions should then be less than the reduction in race.

The following is a rough overview of the experimental design (for details, see method section): In the conditions featuring team membership, an antagonistic encounter between the members of two sports teams was presented. On each team there were either an equal number of Black and White or male and female players. Team membership could be inferred based on both verbal cues (i.e., what each player said) and also visual cues (shared shirt colors). The conditions measuring baseline levels of race and sex categorization were as closely matched to these team conditions as possible—featuring the exact same photo stimuli (i.e., the same faces and shirt color differences) and closely-matched statements. However, all statement content that would indicate that these were two different sports teams interacting was removed (see SOM for details). Finally, within the conditions featuring team membership, there were additional manipulations of both the nature of the sport (either basketball or soccer/European football) and the strength of the team membership (alliance) cues present during the categorization task (see below for details). These additional conditions provided both a within-study replication and a more fine-grained measurement of the effect of crossing race and sex with team membership.

## Results

**Figure 2:**
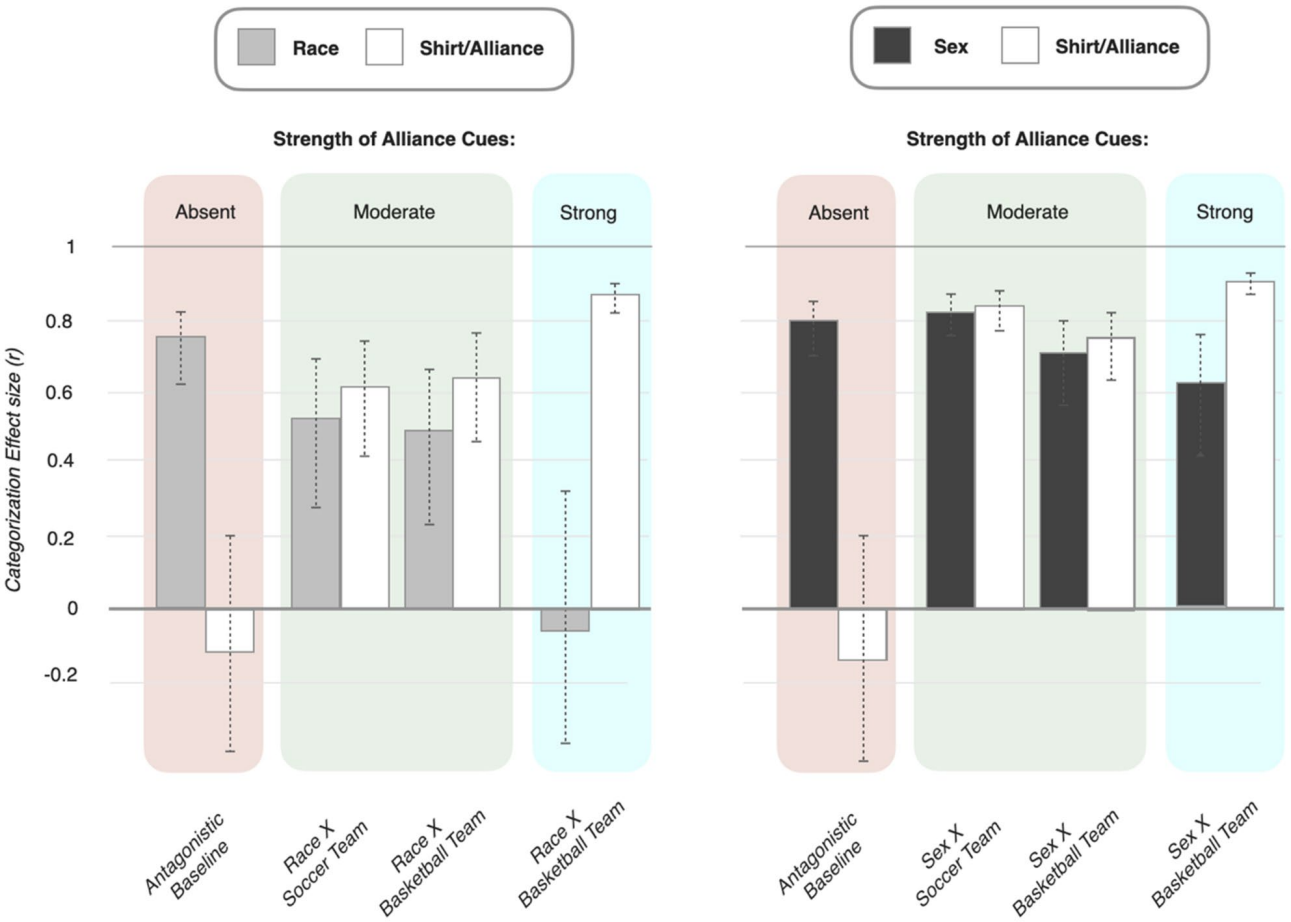
Categorization effect sizes by both race and sex when crossed with alliance membership (in different sporting contexts), using standard t-tests and reported as *r*’s. Error bars are 95% confidence intervals.

The results of four between-subjects conditions featuring race, and another four between-subjects conditions featuring sex, are presented in Fig. 2. (For ease of comparison with past work, differences in means are reported using t-tests and related effect sizes; see SOM for full reporting, including means. Bayesian estimates of the differences in means^29^ were also conducted and are reported in Fig. 4 and fully in the SOM. In all cases, both kinds of analysis lead to the same conclusions).

The left-most condition, *antagonistic baseline*, provided the critical baseline measurement of race and sex absent any alliance information. In this condition there was very strong categorization by both race (*r* = 0.75) and sex (*r* = 0.80), and no appreciable categorization by shirt color. (Shirt color in this baseline condition signified nothing, and was included so that shirt color differences would not be confounded with alliance information across conditions. In fact, for thoroughness—and to unconfound the effect of adding alliance information from the effect of adding antagonism to the study stimuli—two baseline conditions were: One in which targets spoke about positive-to-neutral events (*positive baseline*), and a second in which they spoke about antagonistic events (*antagonistic baseline*). For simplicity, only the antagonistic baseline is presented here, as it is the minimal pair with all three alliance conditions. Notably, there was no appreciable change in categorization by either race (∆ *r* = 0.04) or sex (∆ *r* = 0.02) across these two different baselines, although categorization by race did increase descriptively when antagonism was added (*positive r* = 0.69, *antagonistic r* = 0.75), in contrast to sex (*positive r* = 0.84, *antagonistic r* = 0.80; see SOM for full results)).

The crucial question was what would happen when race and sex are crossed with team membership—the experimental manipulation that should (1) engage the mind’s alliance-tracking leading to categorization by team and (2) that should differentially reduce categorization by race compared to sex. Results are presented in the three conditions to the right of the baselines in Fig. 2 (two featuring moderate strength alliance cues, and one strong alliance cues; see Box 2 for explanation).

**Box 2:**
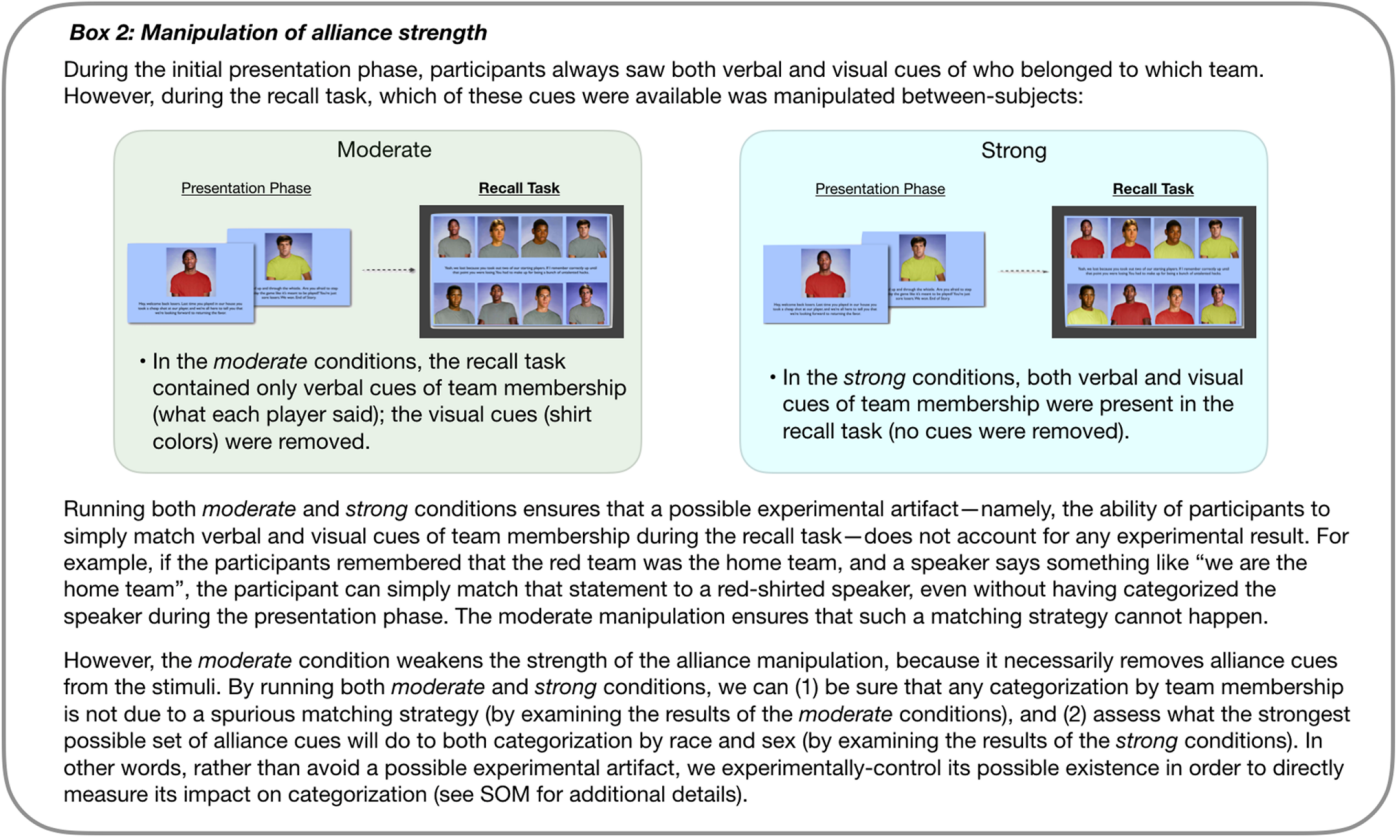
Manipulation of alliance strength.

As can be seen, there was substantial categorization by team membership (*shirt/alliance*) in all three conditions. Importantly, this categorization cannot simply be attributed to the visual salience of the shirt colors—as the exact same visual stimuli were present in the baseline conditions, but elicited no categorization. Thus, participants were keeping track of and categorizing each target by their respective team membership—the first prediction of the alliance hypothesis.

The second and most important prediction of the alliance hypothesis is the differential reduction in race. As can be seen, compared to the baseline condition, categorization by race was reduced more than was sex, and this was true across all three alliance conditions. To fully unpack this result it is helpful to understand what each of the three alliance conditions were.

In all three, either race or sex was crossed with verbally and visually-marked team membership. Two of the three conditions featured *moderately-strong* alliance cues in the stimuli (in which the visual cues of team membership were removed during the recall task), whereas the third and last condition featured *strong* alliance cues in the stimuli (in which the visual cues of team membership were retained during the recall task). This variation in the strength of alliance cues provided both a within-study replication and a more continuous measurement of the effect of alliance cues on race and sex (see Box 2 and SOM). Roughly speaking, the true effect of the alliance manipulation can be understood as lying somewhere between the *moderate* and *strong* conditions. Finally, one additional condition featuring soccer (European football) was also included to ensure that there were no idiosyncratic effects of a basketball sporting context (which was all that was used in Kurzban et al.).

As depicted in Fig. 2, participants in the *moderate* alliance strength *soccer* condition categorized by race (*r* = 0.53), but at a substantially-reduced level compared to baseline (∆ *r* = 0.38). The *moderate basketball* condition produced a nearly-identical result (*r* = 0.49, ∆ *r* = 0.39). Finally, participants in the *strong* alliance strength condition (featuring basketball teams) did not even categorize by race at all (*r* = -0.07, ∆ *r* = 0.54).

The results for sex were markedly weaker or non-existent. Participants in the *moderate* alliance strength *soccer* condition in fact categorized by sex even slightly *more* than compared to baseline (*r* = 0.82, ∆ *r* = 0.38). In the *moderate basketball* condition participants again strongly categorized by sex (*r* = 0.71). However, this time the level of categorization was slightly, but detectably, lower than that found in the baseline condition (∆ *r* = 0.27). Finally, participants in the *strong* alliance strength condition (featuring basketball teams) continued to categorize by sex at high levels (*r* = 0.62). This level of categorization did reflect a lowering of categorization compared to baseline condition (∆ *r* = 0.41), but not compared to the *moderate basketball* condition (∆ *r* = 0.18). Thus, both basketball conditions (that this, both the moderate and strong conditions) did reduce categorization by sex to some degree; the reduction being somewhat lower in the *strong* condition. Finally, substantial categorization by team was found—replicating the effects found in the race conditions.

**Figure 3:**
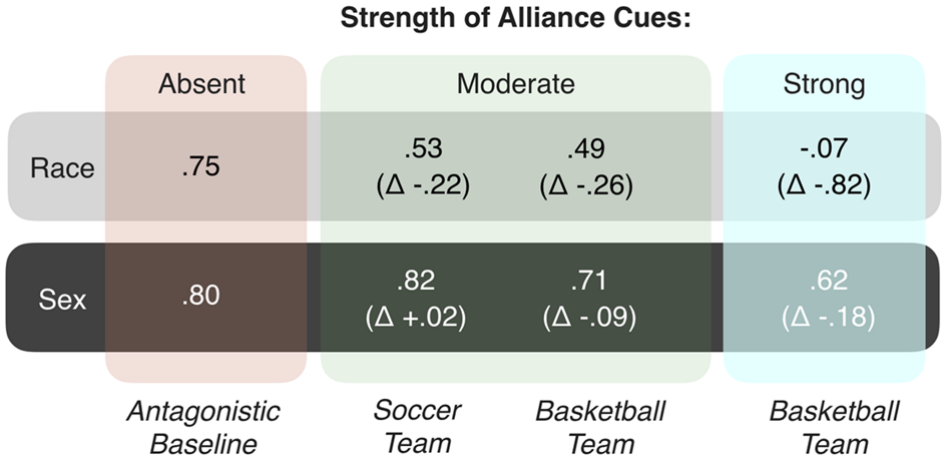
Categorization effect sizes (*r*) for race and sex across the baseline and alliance conditions, with changes in categorization (Δ) denoting the difference in magnitude compared to baseline.

**Figure 4:**
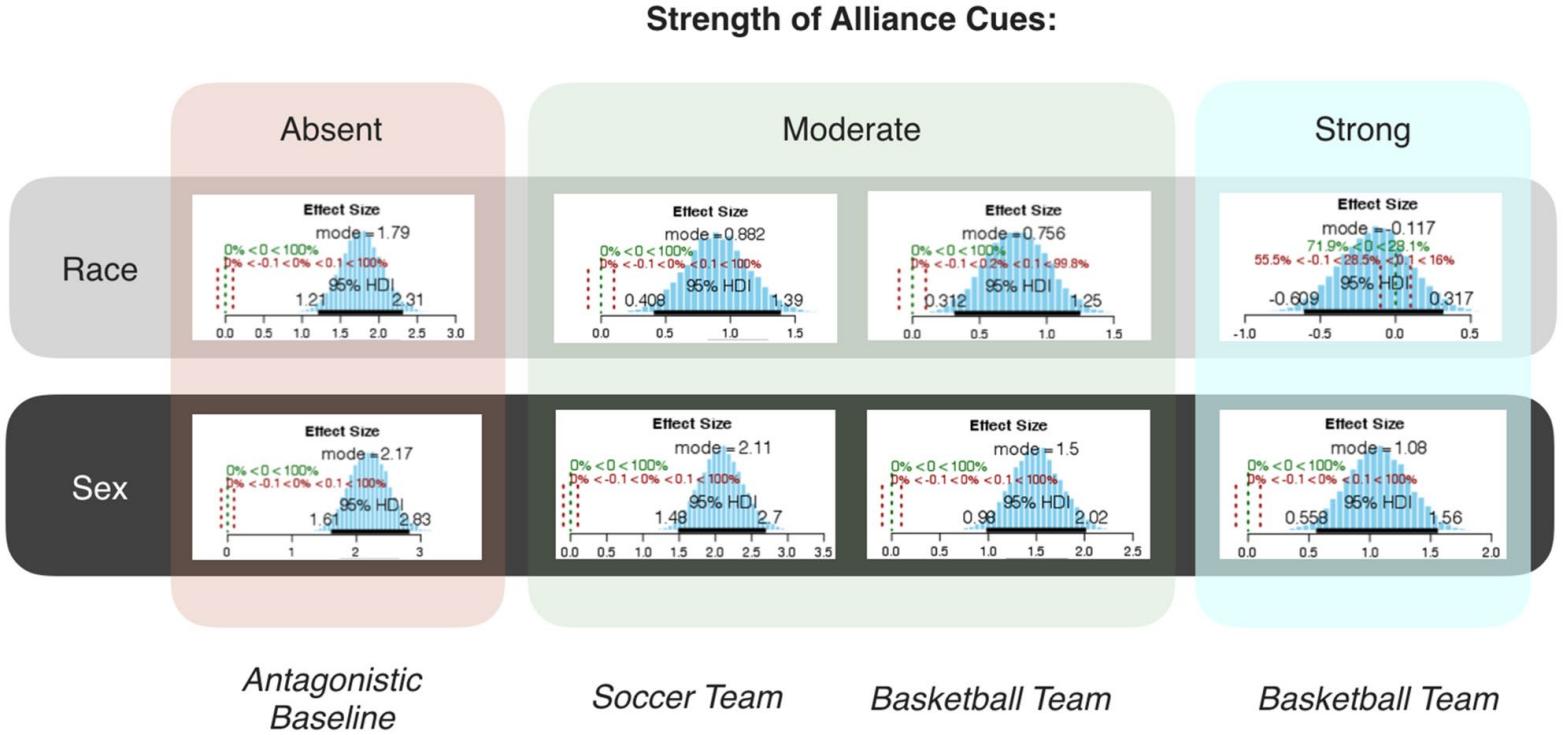
Posterior distributions of categorization effect sizes, using Bayesian estimates of differences in means.

A summary of the changes in effect sizes across the different conditions for race and sex is depicted in Fig. 3. As can be seen in Fig. 3, a decrease in categorization by sex (i) did not occur at all in the *moderate soccer* condition, (ii) was 1/3rd the magnitude of the decrease in categorization by race (Δ *r’s* = 0.09 vs. 0.26, respectively) in the *moderate basketball* condition, and (iii) was 1/5th the magnitude (Δ *r’s* = 0.18 vs. 0.82, respectively) in the *strong basketball* condition. Figure 4 shows the same results for a Bayesian estimate of categorization effect sizes. As can be seen, the same overall pattern of results is found (see SOM for full report).

In sum, these results unambiguously support the predictions of the alliance hypothesis. Namely, that (1) categorization by a now-relevant alliance membership (team membership) will occur, and (2) racial categorization will be differentially lowered when it is shown to be no longer predictive of this alliance membership. Both effects replicated across each of the three team/alliance conditions.

## Discussion

Why people categorize others by their race is a fundamentally-important scientific problem with far-reaching sociological implications. The studies reported here provide the first adequate test of the alliance hypothesis of race—the hypothesis that racial categorization is a modifiable byproduct of alliance tracking—in the context of conflict. Given the long-standing evolutionary history of multi-agent conflict in our own and closely-related species^30^ any hypothesis appealing to an evolved psychology of alliances must survive a test of its predictions under conditions of intergroup conflict. Here, we find that the alliance hypothesis of race in fact meets that standard.

The implications of this finding are far-reaching. First, it provides direct, experimental evidence for a particular causal account of where racial categorization comes from. Namely, that people categorize other people by their race (at least in part) because of exposure to social ecologies in which certain physical features correlate with or track patterns of segregation and discrimination. That is, if one happens to experience that particular physical features correlate with who tends to interact with whom, live with whom, and so on, then these patterns will get picked up by the minds’ alliance-tracking. It is then the operation of this alliance-tracking mental machinery (likely in concert with other mechanisms) that eventually delivers the impression that there are categories of people based on physical features.

Second, and more fundamentally, this finding suggests that racial perception and categorization is in a deep and fundamental way not about attention to physical features per se, but rather is really about predicting social relationships. In other words, out of all the ways in which people may look different from one another, only a subset of these differences become imbued with social relationship relevance over the course of one’s lifetime, and it is those differences that become the basis of automatic categorization. That is, race is a dimension of social categorization because of its social reality—not because of its biological reality or visual salience^24,31–34^. While this idea has historical support—in that the physical features considered “racial categories” have changed over historical time, as one would expect if it were a social construct^31,32^—it can also be directly tested within the mind. The social construction of race in the world, in other words, leaves its mark on the information-processing within the mind. Here, we find evidence for the social reality, but otherwise arbitrary nature, of racial categories.

The current studies are also notable because they provide an adequate test of a hypothesis presented nearly 20 years ago. The paper presenting this hypothesis—Kurzban et al.^6^—is incorrectly but more-or-less universally-cited as providing evidence in support of that hypothesis. Here, we have shown why this is not the case. The good news is that based on the current findings we can see that Kurzban et al. were ultimately correct about their hypothesis. The bad news is that it took nearly 20 years for anyone to notice that the design of that paper did not provide adequate evidence in support of it.

It is a testament to the alliance hypothesis and the evolutionary framework that generated it that its predictions have stood up to experimental scrutiny nearly two decades later. But it is no compliment to the wide variety of psychological and behavioral sciences that had consumed the original Kurzban et al. finding that the inadequacy of that experimental design was missed for so long in such a high-profile paper. This should be cause for concern.

**Figure 5:**
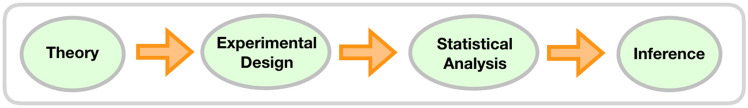
In the process from going from theory to inference using empirical methods, considerable attention has been paid to issues surrounding statistical analysis (step three), but considerably less to the adequacy of experimental design (step two).

Indeed, while statistical analysis and its reporting have received considerable attention lately in light of the replication crisis—with a corresponding focus on the question of “does the experimental effect replicate?”^22^—far less attention has been paid to the more important issue of experimental design and its applicability to the theory that it was set up to test in the first place (see Fig. 5).

The issue of experimental design is critical for understanding exactly why we cannot—and should not have—made any strong inferences based on the results of Kurzban et al. Leaving aside the issue of the biased error correction method, and thus ignoring our uncertainty about the accuracy of the numbers reported (see Introduction), a reasonable reader may wonder why we cannot use the fact that race was reduced in Kurzban et al. as a demonstration of the validity of the alliance hypothesis of race. The problem is that we cannot know *why* race was reduced in Kurzban et al. because in that study there was a failure to experimentally-isolate the effect of crossing race with alliance membership from the effect of adding shirt color to the stimuli. Here, in contrast, because the non-alliance baseline condition also featured differences in shirt color, we can be certain that our effects were only due to the alliance membership manipulation, and not caused by shirt color differences.

Moreover, because we *did* also manipulate the presence or absence of shirt color differences within the alliance conditions (at recall) here, we can directly assess if shirt color differences *do* have an additional effect on racial categorization—when (and only when) they mark alliance membership. We find here that they do. Thus, based on our results, we can speculate that perhaps the reduction in race found in Kurzban et al. was after all due to alliance cues in the stimuli—although we can never be certain of this because those results are contaminated by (1) the biased error correction method and (2) a matching strategy that can lead to an across-the board reduction in both race and sex as a result of an experimental artifact (see Box 2 and SOM), both of which may have fully accounted for their results. Thus, the best we can say for the Kurzban et al. study is that it was the source of an important and subsequently supported hypothesis, but did not provide adequate evidence in support of it.

Of course, it would be remarkable if the first attempt to empirically-validate a new hypothesis turned out, in the fullness of time, to be the soundest evidence in support of it. The current situation just happens to be a particularly extreme case. Notwithstanding that adequate and necessary tests of the hypothesis have taken some time to establish, with the inclusion of the results found here and elsewhere, the alliance hypothesis is beginning to collect a substantial evidentiary basis. Indeed, converging lines of evidence, from multiple methodologies, are consistent with the idea that the mind did not evolve to categorize others by their race^12,35,36^ ("race” being an arbitrary and socially-contingent construct in any case). Rather, race appears to be an instance of a broader, more-general evolved entry in the human mind—one (at least in part) concerned with tracking patterns of alliance and affiliation^14,17,18,20,33,37–40^. In other words, although a seemingly self-evident feature of the world, race is in fact a highly-abstract social construction, built by our evolved cognition when operating within specific kinds of social environments. In particular, if and when certain physical features become imbued with alliance-relevant information (i.e., they predict who interacts with whom, who lives with whom, etc.), then these will come to be treated as important social cues by the mind, and will be perceived as psychologically-salient. For this reason, when the hypothesized inputs to the mind’s alliance-tracking systems are satisfied in the form of clearer information about who is allied with whom, the psychological relevance of race dissipates. Such findings—capped off by the present studies—suggest that we might next aspire to do more than simply erase race, but rather more deeply understand it.

## Method

### Design

All alliance conditions featured an antagonistic encounter between the members of two sports teams, in which either race or sex (varied between-subjects) was crossed with visually and verbally-marked team membership. That is, on each team there were an equal number of Black and White or male and female players. Categorization by both team and the cross-cutting dimension (either race or sex) was measured (how will be explained below).

Non-alliance baseline conditions provided the critical measure of categorization by race and by sex absent any cross-cutting team membership. These baseline conditions were as closely matched to the alliance/team conditions as possible, including using the exact same photo stimuli (i.e., the same faces and shirt color differences) and closely-matched statements. However, all content that would suggest to participants that these were two different sports teams was removed. Thus, participants simply observed individuals making random statements stripped of any alliance cues (see SOM for statements).

There were two of these baseline conditions. One featured positively-valanced statements. The other featured antagonistic statements. Including both was necessary because in the alliance conditions, antagonism and team membership are both present. That is, team members are discussing conflict and fighting. Therefore, if one were to only compare the team/alliance conditions to a neutral baseline condition, one could not establish if any change in categorization by either race or sex was due to either the team/alliance membership in the statements, or to the antagonism in the statements. There are good reasons to be concerned about this. For example, it may be that antagonism prompts attention to race, but drops attention to sex. A neutral to positively-valanced baseline condition was therefore included to first establish baseline levels of categorization by race and sex absent both antagonism and team membership. A second, antagonistic baseline was then also included to compare to this positive baseline. This antagonistic baseline then served as a valanced-matched comparison to the alliance/team conditions.

Three different versions of the alliance/team conditions were run. In all three, race or sex was crossed with team membership. Two of the three conditions differed in the sport being presented, but were otherwise identical. One of these featured basketball teams, following Kurzban et al. The other featured soccer/European football teams. Varying the sporting context provided a within-study replication and ensured that there was nothing idiosyncratic about the basketball team context.

The third and final alliance/team condition was designed to directly measure the potential impact of an experimental artifact that would have allowed participants to simply match verbal and visual cues of team membership during the recall task (see Box 2 and SOM). Past research suggests that this artifact will likely over-inflate the true effect of categorization by team, and may produce a small, domain-general decrease in categorization by the crossed category^17,41^. Here, rather than avoiding the artifact, it was intentionally produced in this one condition. Thus, rather than speculating about what might happen if this artifact is present, we can directly assess what happens experimentally (for details of how this artifact was implemented in this third condition, and prevented in the other two alliance/team conditions, see Box 2 and SOM).

All five conditions—the two baselines and three alliance conditions—were presented within either in a context in which targets differed in race or in sex. Thus, ten between-subjects conditions were run in total.

### Paradigm

All conditions were run using the memory confusion paradigm^17,26,42–44^. This paradigm measures categorization by capturing patterns of errors in memory—specifically, speaker-attribution errors^28^. In the initial phase of the paradigm, a sequence of photos of individual people is presented. Each photo is accompanied by a statement (appearing in text in these studies). Participants are instructed to form impressions of the speakers. After the presentation of photos and statements (here, eight speakers making three statements each, for a total of 24 statements), a short, unrelated distractor task appears. This is used to avoid recency and rehearsal effects. In the final phase, participants are shown an array all of the speakers, and are asked to assign each statement that they had seen previously to the correct speaker (for full details, see Box 1 and SOM).

Errors rates in this attribution task, which are typically quite high, diagnose categorization: To the degree a participant categorized speakers by one or more shared-dimensions (such as team membership or race or sex), they will be more likely to make within-category errors than between-category errors (for details of the error rate calculation, including a new and improved correction for base-rate probabilities^27,28^). Thus, for each participant, three data points are calculated: (1) total errors, (2) within-category errors, and (3) between-category errors. The effect size of the within- versus between-category error difference quantifies categorization.

### Participants and procedure

Participants were 396 University of California at Santa Barbara undergraduate students (mean age 19.01 years, SD = 1.39; 284 F, 110 M, 2 unspecified). These participated in-person in a computer lab for course credit. A minimum sample size of 33 participants per condition was determined ahead of time based on past studies of this type (typical effect sizes in social categorization research of this type are now well-established based on larger sample-size studies^17^), with the stipulation that additional participants would be collected if available from the subject pool. No data were analyzed until the termination of running. Final participant count per condition ranged between 37–41 participants per condition. Research assistants blind to the details of the conditions were instructed to note if any participants egregiously rushed through the task or were extremely distracted (if they were on their phone, for example). Four out of 400 total participants were flagged according to these criteria, and the data from these four were never analyzed.

After completing the memory confusion task, participants filled out a short demographic sheet, indicating their age and sex. Demographics of two participants could not be determined. No pilot tested was conducted. Data stripped of participant-identification are freely-available as Supplementary Online Materials, as are the instructions and statement stimuli. The study protocol was approved by the University of California at Santa Barbara IRB, and as such the running of the study was performed in accordance with all relevant guidelines and regulations.

Informed consent was obtained from all participants and/or their legal guardians.

## Supplementary Information

Supplementary Information 1.

Supplementary Information 2.

## Data Availability

Data stripped of participant-identification are freely-available as Supplementary Online Materials published alongside this article.
