# Supplementary material for: The correct way to test the hypothesis that racial categorization is a byproduct of an evolved alliance-tracking capacity
Source: Sci Rep. 2021 Feb 9;11:3404. doi: 10.1038/s41598-021-82975-x (PMC7873069; doi:10.1038/s41598-021-82975-x)

SOM: *The correct way to test the hypothesis that racial categorization is a byproduct of an evolved alliance-tracking capacity.*

David Pietraszewski

Index

Additional details about the alliance strength manipulation……….………………………..…p. 2-3

Methodological details of the memory confusion paradigm………………….….…..……..p. 4

Introduction & Instructions……………………………………………………..…..…..…p.5-6

Statements……………………………………………………………………..………p.7-13

Full descriptive statistics…………………………………………….………………..p.14-17

Bayesian analysis……………………………………………….….….……..……p.18-43

Additional Details About the Alliance Strength Manipulation

The paradigm used in this paper—the memory confusion paradigm—relies on patterns of attribution errors to infer psychological categorization. For example, if participants categorize targets by their sports team membership then they will be more likely to attribute what one team member said to another member of the same team (see Box 1 of main text).

An issue arises, however, when information about team membership is present in both the photos of the targets (for example, marked by what they wear) and also in the statements themselves (for example, if the speaker says something about “We are the home team”; see Box 2 of main text). The problem is then that during the recall task, participants may be able to simply match visual and verbal cues. For example, if the participants remembered that the red-shirted targets were the home team, then seeing a statement that contains the phrase “We are the home team” would allow the participant to assign that statement to the same team that in fact said that statement. Such a matching strategy would produce a pattern of errors that looked like categorization by team, even if none existed. (In an extreme case, one might be able to guess the team membership correctly even without ever having seen the targets, if the statements themselves can be obviously-matched to one team or the other).

Past work in this paradigm has dealt with this potential matching strategy in a number of different ways[17,18]. One method is provide both verbal and visual cues of team membership during the initial presentation phase, but then to remove one of the two pieces of information during the statement attribution task. This removal makes matching impossible. For example, statement portions that are identified as diagnosing team membership can be removed at recall. Conversely, the visual marker of team membership can be removed (i.e., if targets were wearing different color shirts during the presentation, these are changed to all gray shirts during the statement attribution task). Past work has established the validity of both methods.

Here, both the soccer and basketball conditions feature statements that have weak but appreciable team-diagnosticity (for example, a statement indicating one is a member of the home team that had had a player injured last year, as mentioned during the introduction passages given to participants; see instructions and statements within this SOM for details). Thus, in both conditions removing one source of team-diagnosticity is required during the statement attribution task. This was done by removing the visual marker of team membership: changing the different colored shirts to all gray[18]. The conditions in which this was done are referred to as the *moderate* alliance cue strength conditions in the paper. In order to make the baseline conditions match these experimental conditions as closely as possible, the same shirt-color-to-gray manipulation occurred in those conditions, as well (even though, given the neutral statement content, would not have been strictly necessary). These baseline conditions are referred to as *absent* alliance cue strength conditions in the paper.

The third alliance/team condition, in contrast, was designed to allow this possible matching of verbal and visual cues of team membership. Past work[17] has shown that manipulations of the number of team cues at recall are treated psychologically as cues of the relative importance of that team membership. In other words, removing team-diagnostic statements serves to reduce the cue-strength of team membership. Consequently, both of the conditions in which the matching artifact is prevented are likely to be slight underestimates of

the true effect of team membership. By allowing both verbal and visual cues of team membership at recall, we are seeing the effects of the strongest possible manipulation of cross-cutting team membership[38]. In this way, we can bracket the true effect of crossing team membership with race (or sex) as in-between that which is observed in the matching-prevented and in the matching-allowed conditions. The condition in which this matching was allowed is referred to as the *strong* alliance cue strength condition in the paper (see also Box 2).

Methodological Details of Memory Confusion Paradigm

During the initial presentation phase, shirt colors were yellow and red. Photos in the race conditions featured young men (as past work suggests that these are most strongly categorized by race[17]), and in the sex conditions, all-white young men and women. As in past work, all four combinations of team/shirt color and race/sex were presented during the first four photo presentations (done to establish the orthogonal relationship between the two dimensions as quickly as possible). Red shirt color was always presented first, followed by yellow second, and alternating thereafter. In the race conditions the first two photos were Black targets, followed by two White targets. Targets were randomly matched to statements thereafter, with the stipulation that each target would say three statements in today across all 24 statements. Assignment of individual targets to team was randomized across subjects, within the stipulated constraint that each team must have an equal number of Black and White (or male and female) members. Each photo/statement pairing advanced after 20 seconds.

The distractor task featured a map of the United States, and participants were asked to try to recall as many state capitals as possible. The task auto-terminated after one minute.

In the recall task, all eight targets seen previously were displayed in a randomized array. Each of the statements seen previously then appeared in random order, one at a time. Participants indicated the speaker by clicking on their photo.

Prior to calculating the within- versus between-category error difference, a correction for the different probabilities of these two different error types had to be applied (as the correct speaker cannot count as a within-category error, between-category errors are more likely than are within-category errors in this paradigm.) Here, the new and improved error correction was used — which involved multiplying the between-category errors by .75 once these were aggregated for each participant[27,28].

Introduction & Instructions

Study-Relevant Content in Consent Form:

This study is investigating how people form impressions of others. You will read a hypothetical scenario, be shown a series of images, and be asked to make a number of decisions. This should take about 30 minutes.

Positive Baseline & Antagonistic Baseline Introduction and Instructions:

Several years ago, the U.S. Government wanted to establish a truly random sample of all United States citizens for survey purposes. A lottery system with social security numbers was used. Out of all the citizens in the United States, a small random sample was drawn. These individuals were contacted by local government agencies. To ensure full participation, the government offered a seven year break from all taxes. Participation rates reached 99%. Part of the survey involved collecting several standardized photographs of each participant, a recorded interview, and the completion of several surveys.

You are about to see a sample of this random sample of people. Along with their photograph, you will see a portion of what they said in their interview. The people are not talking to one another. Each statement is an independent sample from each person's pre-recorded interview. To ensure confidentiality of their statements, the government has only released "decontextualized" portions of their statements, so that no personal information is made public. It is these "decontextualized" statements that you will be seeing. In particular, you will be seeing each person talking about some event in their life that had involved severe conflict and antagonism.

We are interested in your impressions of these individuals as they make their statements. You will see their photographs paired with what they said.

The photos you will be seeing will advance automatically, without your having to press anything. After you have completed viewing the photos and statements, you will receive further instructions on the computer screen. If you have any questions, you may ask them now. Otherwise, click on "OK" to begin.

Basketball Conditions Introduction and Instructions:

Several years ago, there was an unfortunate incident involving two basketball teams, one of which had a reputation for playing rough. Toward the end of the second half tempers were running high. The score was extremely close, and there had been a history of rivalry between the two teams. The game had been what sports announcers usually refer to as 'extremely physical'. At one point, one of the players from the home team went down and had to be hospitalized. The referees hadn't seen what had happened, but some players claimed to have seen a member of the visitor's team hit a player as they were making a move towards the basket. In a rush, both teams'

benches emptied, and a fight broke out. It took security several minutes to restore order and, in the end, another player from the home team was also injured in the scuffle.

As punishment for the incident, both teams were put on probation. Far from settling the issue, this led to hard feelings on both sides. However, the teams' schedule calls for them to play each year, and so, the next season, they met again. As part of an attempt at reconciliation, a number of players from each team were put together to talk before the game. This conversation was recorded, and you will be seeing a portion of it.

We are interested in the impressions that these players make on you as they have their discussion. You will see their photographs paired with what they said.

The photos you will be seeing will advance automatically, without your having to press anything. After you have completed viewing the photos and statements, you will receive further instructions on the computer screen. If you have any questions, you may ask them now. Otherwise, click on “OK” to begin.

<u>Soccer Conditions Introduction and Instructions:</u>

Several years ago, there was an unfortunate incident involving two soccer teams, one of which had a reputation for playing rough. Toward the end of the second half tempers were running high. The score was extremely close, and there had been a history of rivalry between the two teams. The game had been what sports announcers usually refer to as 'extremely physical'. At one point, one of the players from the home team went down and had to be hospitalized. The referees hadn't seen what had happened, but some players claimed to have seen a member of the visitor's team hit a player as they were making a move towards the goal. In a rush, both teams' benches emptied, and a fight broke out. It took security several minutes to restore order and, in the end, another player from the home team was also injured in the scuffle.

As punishment for the incident, both teams were put on probation. Far from settling the issue, this led to hard feelings on both sides. However, the teams' schedule calls for them to play each year, and so, the next season, they met again. As part of an attempt at reconciliation, a number of players from each team were put together to talk before the game. This conversation was recorded, and you will be seeing a portion of it.

We are interested in the impressions that these players make on you as they have their discussion. You will see their photographs paired with what they said.

The photos you will be seeing will advance automatically, without your having to press anything. After you have completed viewing the photos and statements, you will receive further instructions on the computer screen. If you have any questions, you may ask them now. Otherwise, click on “OK” to begin.

Statements

Statements Used in Positive Baseline Conditions:

1. They said "Hey, welcome back here. Last time you came to my house I had a great time with you, and I'm here to tell you that I'm looking forward to returning the favor."
2. I didn't know what they were talking about. It was unclear. I got up when I heard a whistle. They were hesitant to step up and act like they meant it. Just sorting things I think. End of story.
3. They lost it at that point. It was one of their biggest problems. If I remember correctly up until that point they hand't lost it. You have to hand it to them for turning back.
4. I was in my place with my friends, and they were getting out of the way. I slept like a baby. There wasn't any conflict.
5. "You're back now. And we'll see what happens today and if we can actually go a full day without the thing going until it breaks", they said. Seriously, what's the point of spending time on something like that if it never turns out right?
6. And what's the point of stacking them if none of them could stand up on their own when someone just brushed into them? Some of that stuff was so fragile that it crumbled if you even looked at it the wrong way.
7. We weren't going to deny that we were throwing things into the pile for them last time. They tripped and fell over when the others weren't looking.
8. Nah. I didn't reply at all. I let my actions speak louder than my words. I had a good plan. Their plan was really complicated.
9. Theirs? They would try and then fail, and then start kicking and swim. I decided that the next time they had even the least amount of trouble with it, I would go help them out the rest of the time.
10. They said "Oh really? You're gonna go with us? You and all of your friends (and I'm counting all three of them), are gonna have a great time going over there, and I think you're gonna like it."
11. I heard some people. It was the sound of their friends outside. Someone told me how many there were when they went out and looked. Then they said, "Hey, give them what you just gave to me, and see how they like it."
12. I loved it. They never complained about things being tough, but they were tough, but only when they needed to be. One hundred percent not just talk. I wish I could have gone back there. Having to leave that place sucked.
13. I really did want them to come to visit me in the first place. They shouldn't have even been in that place at all. I think the last time I saw them I was like a fifth-grader. I said "Why don't you come back here?"
14. They said "We'll be heading down, no problem. But not until we make sure you and your friends are okay. Did you bring a first aid kit? You're gonna need it."
15. It was great. One of my friends was in the hospital when they came in and helped them. "What is this? I didn't expect you to help out with this. I was given lots of warnings last time. It would have been so easy to have just given up on me", they said.

16. They were gonna paddle to me. I was shaking all over, I had the same experiences they all did. And they were the only ones who seemed to be able to help me. What does that say about them?
17. I said "That's not what I hear. I talk all the time with other people about how you can find cheap deals whenever you want." I'm one of the few that actually looked into it.
18. Yeah, and that post-party place cost all of us a lot to get into. We sat there and complained about our jobs during the day, and then it was their friends that kept things going after the party was over. Yeah, I had fun, and they made it a good night.
19. Okay. So they worried about us not being able to handle how they were acting, but when I responded back, they were good about it, and it didn't ruin anything. I was defending my friends. And I wouldn't have needed to do anything if they had just acted like civilized people.
20. "Forgot you?" they said, "I won't ever be accused of anything other than being your best friend all last year. Really glad you're here. I'm gonna head back to my place and introduce you to all my new friends."
21. Yeah, they did a good job of avoiding embarrassment. I told them "Watch it out there. If the wind starts whipping around again, you're gonna get rained out. And then you better get back on the bus and stay there. You don't want to stick around any longer than you need to."
22. That's right. One of them landed on me as I was sitting down. And yeah, I didn't want to leave any sooner than I needed to. I'm glad to hear other people agree with me that that place is really great.
23. They said "I'm going to repay you sometime for really helping out my friend. And I'm going to make sure that you're respected on your own home turf."
24. Blah blah blah. If I got points for flapping my mouth, I'd definitely stay with the entire thing and then some. But it's not worth my time any more. No more, I'm out of there.

<u>Statements Used in Antagonistic Baseline Conditions:</u>

1. They said "Hey, welcome back loser. Last time you came to my house you took a cheap shot at me, and I'm here to tell you that I'm looking forward to returning the favor."
2. I didn't know what they were talking about. It was nonsense. I got up when I heard a whistle. They were afraid to step up and act like they meant it. A sore loser I think. End of story.
3. They lost it because I pointed out two of their biggest problems. If I remember correctly up until that point they hand't lost it. You have to hand it to them for being untalented hacks.
4. I was in my place with my friends, and they still wouldn't get out of our way. They acted like babies. Couldn't handle any conflict.
5. "Well, you're back now. And we'll see what happens today and if you can actually go a full day without things turning into a brawl", they said. Seriously, what's the point of spending time with someone if all they want to do is fight?
6. And what's the point of standing up for yourself if you can't even stay up on your own two feet when someone just brushes into you? Some people are so fragile that they crumble if you look at them the wrong way.

7. They weren't even going to deny that they were throwing elbows into my face all last time. Tripping me. Knocking me over when the others weren't looking.
8. Nah. I didn't deny anything. I let my actions speak louder than my words. I had a good plan. Their plan was complain, fail, repeat.
9. Theirs? They would try and then fail, and then start kicking and punching. I decided that the next time they made even the least amount of contact with me, I would go after them all the rest of the night.
10. They said "Oh really? You're gonna start with us? You and whose army? You and all of your friends (and I'm counting all three of them) are gonna have to deal with us, and you're gonna like it."
11. I heard some people. It was the sound of their friends outside. Someone told me how many there were when they went out and looked. They were yelling "You tell them what you just said to me, and see how they like it."
12. I loved it. They always complained about me being tough, but then also tried to act tough, but only when they outnumbered me like one hundred to one. All talk. I shouldn't have even bothered going back there. That place sucked.
13. Hey I didn't want them to come to visit me in the first place. They shouldn't have even been allowed to be in that place at all. They were acting like a fifth-grader. I said "Why don't you go back to where you came from?"
14. They said "We'll be calming down, no problem. But not until after we trounce you and your little friends. Did you bring a first aid kit? You're gonna need it again."
15. It was all so messed up. One of our friends was in the hospital and then they came in and threatened us. "What is this? We don't need to put up with this. You were all given warnings after last time. It would be so easy to have you all expelled", I said.
16. They were gonna taddle on me. I was shaking all over, I had the same experiences they all did. And they were the only ones who seemed to have a problem with me. What does that say about them?
17. I said "That's not what I hear. I talk all the time with other people about how you take cheap shots whenever you can." I'm one of the few that actually did something about it.
18. Yeah, and that post-party brawl cost all of us the ability to get into there. They sat there and complained about my jabs during the day, but it was their friend that started the fight with me after the party was over. Yeah, I took jabs, but they started the fight.
19. Okay. So they bragged about me not being able to handle how they were acting, but when I responded back, they whined about it ruining their shot at making it. I was defending my friends. And I wouldn't have needed to do anything if they had just acted like civilized people.
20. "Forget you" they said, "I don't need to be accused of anything other than beating you last year and being the better person. I'm gonna head back to my place and then embarrass you in front of your friends."
21. Yeah, they did a good job of being embarrassing. I told them "Watch it out there. If your elbows start flying around again, you're gonna get kicked out. And then you better get back on the bus and stay there. You don't want to stick around any longer than you need to.

22. That's right. One of them fell on me as I was sitting down. And yeah, I didn't want to be there any longer than I needed to be. I'm glad to hear people agree with me that that place is terrible.
23. They said "I'm going to make you sorry you ever took out my friend. And I'm going to make you sorry you disrespected me on my home turf."
24. Blah blah blah. If they could win points for flapping their mouth, they'd definitely get the entire thing and then some. But they're not worth my time any more. No more, I'm out of there.

<u>Statements Used in Soccer Conditions:</u>

1. Hey, welcome back losers. Last time you played in our house you took a cheap shot at our player, and we're all here to tell you that we're looking forward to returning the favor.
2. What are you talking about? That's nonsense. We played up and through the whistle. Are you afraid to step up and play the game like it's meant to be played? You're just sore losers. We won. End of story.
3. Yeah, we lost because you took out two of our starting players. If I remember correctly up until that point you were losing. You had to make up for being a bunch of untalented hacks.
4. You were in your own house, with your own fans, and still couldn't pull out the win. Aww, poor babies. Can't handle a little contact.
5. Well, you're back now. And we'll see who wins today. And we'll see if you can actually go a full game without turning it into a brawl. Seriously, what's the point of playing a skill sport if all you want to do is fight?
6. And what's the point of showing up for a soccer match if you can't stay up on your own two feet when someone brushes into you? Your players are so fragile that they crumble if you look at them the wrong way.
7. So you're not going to deny that you were throwing elbows into our faces all last time? Tripping us? Knocking us over when the refs weren't looking?
8. Nah. We don't need to deny anything. We let our actions speak louder than our words. We've got our gameplan. What's yours? Complain, lose, repeat?
9. And what's yours? Fail at soccer and then start kicking and punching? We've decided that if any of you make even the least amount of contact after the whistle, we're gonna go after you all the rest of the night.
10. Oh really? You're gonna go after us? You and whose army? You and all of your fans (and I'm counting all three of them here) are gonna have to deal with another loss, and you're gonna like it.
11. You hear that hum? That's the sound of our fans out there. You tell me how many there are when you step out there. There's more than three. You tell them what you just said to us, and see how they like it.
12. I love it. You all complain about us playing rough, but then also try to act tough, but only when you outnumber us like one hundred to one. All talk. We shouldn't have even bothered coming back here. This place sucks.

13. Hey we didn't want you to come here to play us in the first place. You shouldn't even be allowed to play in this division at all. You play at a fifth-grade level anyhow. So why don't you go back to where you came from?
14. We'll be getting going, no problem. But not until after we trounce you and all your little friends. Did your trainer bring the first aid kit? You're gonna need it again.
15. You are all so messed up. One of our players was in the hospital last time and you come in here and threaten us? What is this? We don't need to put up with this. You were all given warnings after last time. It would be so easy to have you all ejected.
16. Hey, get this, they're gonna taddle on us. We play all over, we play the same teams you all do. And you're the only ones who seem to have a problem with us. What does that say about you?
17. That's not what we hear. We talk all the time with other teams about how you all take cheap shots whenever you can. We're one of the few teams that actually did something about it.
18. Yeah, and that post-play brawl cost all of us the ability to get into the playoffs. You sit here and complain about our elbows during play, but it was your team that started a brawl with us after the play was over. Yeah, we play physical, but you started the fight.
19. Okay. So you brag about us not being able to handle your physical game, and then when we respond back, you whine about it ruining your shot at the playoffs? We were defending our players. And we wouldn't have needed to do anything if you had just played like civilized people.
20. Forget this. We don't need to be accused of anything other than beating you last year and being the better team. We're gonna head back to our locker room and then embarrass you all in front of your fans.
21. Yeah, you all sure do a good job of being embarrassing. Just watch it out there. If your elbows start flying around again, you're gonna get kicked out. And then you better get back on the bus and stay there. You don't want to stick around any longer than you need to.
22. You're right. One of you may fall on us if we stick around. And yeah, we don't want to be here any longer than we need to be. I'm glad to hear you agree with us that this place is terrible.
23. We are going to make you sorry you ever took out our players. And we're going to make you sorry you ever disrespected us on our home turf.
24. Blah blah blah. If you all got points for flapping your mouths, you'd definitely win the entire division and then some. But you're not worth our time any more. Come on, we're out of here.

<u>Statements Used in Basketball Conditions:</u>

1. Hey, welcome back losers. Last time you played in our house you took a cheap shot at our player, and we're all here to tell you that we're looking forward to returning the favor.
2. What are you talking about? That's nonsense. We played up and through the whistle. Are you afraid to step up and play the game like it's meant to be played? You're just sore losers. We won. End of story.
3. Yeah, we lost because you took out two of our starting players. If I remember correctly up until that point you were losing. You had to make up for being a bunch of untalented hacks.

4. You were in your own house, with your own fans, and still couldn't pull out the win. Aww, poor babies. Can't handle a little contact.
5. Well, you're back now. And we'll see who wins today. And we'll see if you can actually go a full game without turning it into a brawl. Seriously, what's the point of playing a skill sport if all you want to do is fight?
6. And what's the point of showing up for a basketball game if you can't stay up on your own two feet when someone brushes into you? Your players are so fragile that they crumble if you look at them the wrong way.
7. So you're not going to deny that you were throwing elbows into our faces all last time? Tripping us? Knocking us over when the refs weren't looking?
8. Nah. We don't need to deny anything. We let our actions speak louder than our words. We've got our gameplan. What's yours? Complain, lose, repeat?
9. And what's yours? Fail at basketball and then start kicking and punching? We've decided that if any of you make even the least amount of contact after the whistle, we're gonna go after you all the rest of the night.
10. Oh really? You're gonna go after us? You and whose army? You and all of your fans (and I'm counting all three of them here) are gonna have to deal with another loss, and you're gonna like it.
11. You hear that hum? That's the sound of our fans out there. You tell me how many there are when you step out there. There's more than three. You tell them what you just said to us, and see how they like it.
12. I love it. You all complain about us playing rough, but then also try to act tough, but only when you outnumber us like one hundred to one. All talk. We shouldn't have even bothered coming back here. This place sucks.
13. Hey we didn't want you to come here to play us in the first place. You shouldn't even be allowed to play in this division at all. You play at a fifth-grade level anyhow. So why don't you go back to where you came from?
14. We'll be getting going, no problem. But not until after we trounce you and all your little friends. Did your trainer bring the first aid kit? You're gonna need it again.
15. You are all so messed up. One of our players was in the hospital last time and you come in here and threaten us? What is this? We don't need to put up with this. You were all given warnings after last time. It would be so easy to have you all ejected.
16. Hey, get this, they're gonna taddle on us. We play all over, we play the same teams you all do. And you're the only ones who seem to have a problem with us. What does that say about you?
17. That's not what we hear. We talk all the time with other teams about how you all take cheap shots whenever you can. We're one of the few teams that actually did something about it.
18. Yeah, and that post-play brawl cost all of us the ability to get into the playoffs. You sit here and complain about our elbows during play, but it was your team that started a brawl with us after the play was over. Yeah, we play physical, but you started the fight.
19. Okay. So you brag about us not being able to handle your physical game, and then when we respond back, you whine about it ruining your shot at the playoffs? We were defending our

players. And we wouldn't have needed to do anything if you had just played like civilized people.

20. Forget this. We don't need to be accused of anything other than beating you last year and being the better team. We're gonna head back to our locker room and then embarrass you all in front of your fans.
21. Yeah, you all sure do a good job of being embarrassing. Just watch it out there. If your elbows start flying around again, you're gonna get kicked out. And then you better get back on the bus and stay there. You don't want to stick around any longer than you need to.
22. You're right. One of you may fall on us if we stick around. And yeah, we don't want to be here any longer than we need to be. I'm glad to hear you agree with us that this place is terrible.
23. We are going to make you sorry you ever took out our players. And we're going to make you sorry you ever disrespected us on our home turf.
24. Blah blah blah. If you all got points for flapping your mouths, you'd definitely win the entire division and then some. But you're not worth our time any more. Come on, we're out of here.

Full Descriptive Statistics

Race Conditions

Positive Baseline Condition, (N=40)

- total errors: M = 16.17, SD = 3.33
- more within-race errors (M = 9.50, SD = 3.11) than between-race errors (M = 5.01, SD = 2.42), t(39) = 6.01, p < .001*, *r* = .69 (*note that all p values will be two-tailed).
- no more within-shirt-color errors (M = 7.35, SD = 3.11) than between-shirt-color errors (M = 6.62, SD = 1.85), t(39) = 1.13, p = .264, *r* = .18

Antagonistic Baseline Condition, (N=41)

- total errors: 15.27, SD = 3.87
- more within-race errors (M = 9.32, SD = 2.85) than between-race errors (M = 4.46, SD = 2.65), t(40) = 7.07, p < .001, *r* = .75
- no more within-shirt color-errors (M = 6.24, SD = 3.04) than between-shirt-color errors (M = 6.77, SD = 2.53), t(40) = -.75, p = .456, *r* = -.12

Positive vs. Antagonistic Baseline Conditions

- no substantial change in categorization by race (*positive baseline* M = 4.49, SD = 4.73; *antagonistic baseline* M = 4.85, SD = 4.40; t(79) = .36, p = .724, *r* = .04)

Moderate Alliance Strength Soccer Condition, (N=39)

- total errors: M = 17.15, SD = 3.22
- more within-race errors (M = 8.38, SD = 2.24) than between-race errors (M = 6.58, SD = 1.82), t(38) = 3.83, p < .001, *r* = .53
- more within-team errors (M = 9.85, SD = 3.25) than between-team errors (M = 5.48, SD = 3.00), t(38) = 4.84, p < .001, *r* = .62

Moderate Alliance Strength Soccer vs. Antagonistic Baseline Conditions

- substantial decrease in categorization by race compared to baseline (error difference in the *antagonistic baseline*: M = 4.85, SD = 4.40; error difference in the *moderate soccer* condition: M = 1.81, SD = 2.95; change in categorization: t(78) = -3.62, p = .001, r = .38)
- substantial increase in categorization by team compared to baseline shirt color categorization (error difference in the *antagonistic baseline:* M = -.52, SD = 4.46; error difference in the *moderate soccer* condition: M = 4.37, SD = 5.63; change in categorization: t(78) = 4.32, p < .001, *r* = .44)

Moderate Alliance Strength Basketball Condition, (N=40)

- total errors: M = 17.5, SD = 3.73
- more within-race errors (M = 8.48, SD = 2.09) than between-race errors (M = 6.77, SD = 2.29), t(39) = 3.52, p = .001, *r* = .49

- more within-team errors (M = 10.35, SD = 3.48) than between-team errors (M = 5.36, SD = 3.29), t(39) = 5.26, p < .001, *r* = .64

<u>*Moderate Alliance Strength Basketball vs. Antagonistic Baseline Conditions*</u>
- substantial decrease in categorization by race compared to baseline (error difference in the *antagonistic baseline:* M = 4.85, SD = 4.40; error difference in the *moderate basketball* condition: M = 1.71, SD = 3.07; change in categorization: t(79) = -3.73, p < .001, *r* = .39)
- substantial increase in categorization by team compared to baseline shirt color categorization (error difference in the *antagonistic baseline:* M = -.52, SD = 4.46; error difference in the *moderate basketball* condition: M = 5.00, SD = 5.99; change in categorization: t(79) = 4.70, p < .001, *r* = .47)

<u>*Strong Alliance Basketball Condition, (N=39)*</u>
- total errors: M = 15.41, SD = 3.09
- no more within-race errors (M = 6.46, SD = 2.45) than between-race errors (M = 6.71, SD = 1.98), t(38) = -.44, p = .661, *r* = -.07
- more within-team errors (M = 12.54, SD = 3.27) than between-team errors (M = 2.15, SD = 3.15), t(38) = 11.02, p < .001, *r* = .87

<u>*Strong Alliance Basketball vs. Antagonistic Baseline Conditions*</u>
- substantial decrease in categorization by race compared to baseline (error difference in the *antagonistic baseline*: M = 4.85, SD = 4.40; error difference in the *strong basketball* condition: M = -.25, SD = 3.54; change in categorization t(78) = -5.70, p < .001, *r* = .54)
- substantial increase in categorization by team compared to baseline shirt color categorization (error difference in the *antagonistic baseline:* M = -.52, SD = 4.46; error difference in the *strong basketball* condition: M = 10.38, SD = 5.89; change in categorization: t(78) = 9.37, p < .001, *r* = .73)

<u>*Strong Alliance Basketball vs. Moderate Alliance Basketball Conditions*</u>
- substantial decrease in categorization by race compared to moderate (error difference in the *moderate basketball* condition: M = 1.71, SD = 3.07; error difference in the *strong basketball* condition: M = -.25, SD = 3.54; change in categorization: t(77) = -2.63, p = .01, *r* = .29)
- substantial increase in categorization by team compared to moderate (error difference in the *moderate basketball* condition: M = 4.99, SD = 6.00; error difference in the *strong basketball* condition: M = 10.38, SD = 5.89; change in categorization: t(77) = 4.04, p < .001, *r* = .42)

Sex Conditions

<u>*Positive Baseline Condition, (N=41)*</u>
- total errors: M = 14.44, SD = 4.56
- more within-sex errors (M = 9.46, SD = 3.06) than between-sex errors (M = 3.73, SD = 2.43), t(40) = 9.60, p < .001, *r* = .84

- no more within-shirt-color errors (M = 5.83, SD = 2.66) than between-shirt -color errors (M = 6.46, SD =2.51), t(40) = -1.18, p = .245, *r* = -.18

Antagonistic Baseline Condition, (N=40)
- total errors: 15.55, SD = 3.50
- more within-sex errors (M = 10.03, SD = 2.75) than between-sex errors (M = 4.14, SD = 2.65), t(39) = 8.22, p < .001, *r* = .80
- no more within shirt color errors (M = 6.35, SD = 2.57) than between shirt color errors (M = 6.90, SD =2.36), t(39) = -.89, p = .380, *r* = -.14

Positive vs. Antagonistic Baseline Conditions
- no substantial change in categorization by sex (*positive baseline:* M = 5.73, SD = 3.82; *antagonistic baseline:* M = 5.88, SD = 4.52; t(79) = .16, p = .873, *r* = .02)

Moderate Alliance Strength Soccer Condition, (N=39)
- total errors: M = 14.46, SD = 3.42
- more within-sex errors (M = 8.85, SD = 2.51) than between-sex errors (M = 4.21, SD = 1.87), t(38) = 8.96, p < .001, *r* = .82
- more within-team errors (M = 10.38, SD = 2.86) than between-team errors (M = 3.06, SD =2.74), t(38) = 9.51, p < .001, *r* = .84

Moderate Alliance Strength Soccer vs. Antagonistic Baseline Conditions
- no change in categorization by sex compared to baseline (error difference in the *antagonistic baseline:* M = 5.88, SD = 4.52; error difference in the *moderate soccer* condition: M = 4.63, SD = 3.23; change in categorization: t(77) = -1.41, p = .164, *r* = .16)
- substantial increase in categorization by team compared to baseline shirt color categorization (error difference in the *antagonistic baseline:* M = -.55, SD = 3.92; error difference in the *basketball* condition: M = 6.39, SD = 5.72; change in categorization: t(78) = 6.33, p < .001, *r* = .58)

Moderate Alliance Strength Basketball Condition, (N=40)
- total errors: M = 15.22, SD = 3.50
- more within-sex errors (M = 8.60, SD = 2.57) than between-sex errors (M = 4.97, SD = 2.19), t(39) = 6.24, p < .001, *r* = .71
- more within-team errors (M = 10.18, SD = 3.51) than between-team errors (M = 3.79, SD =2.96), t(39) = 7.06, p < .001, *r* = .75

Moderate Alliance Strength Basketball vs. Antagonistic Baseline Conditions
- small but detectable decrease in categorization by sex compared to baseline (error difference in the *antagonistic baseline:* M = 5.88, SD = 4.52; error difference in the *moderate basketball* condition: M = 3.63, SD = 3.68; change in categorization: t(78) = -2.44, p = .017, *r* = .27)

- substantial increase in categorization by team compared to baseline shirt color categorization (error difference in the *antagonistic baseline:* M = -.55, SD = 3.92; error difference in the *moderate basketball* condition: M = 6.39, SD = 5.72; change in categorization: t(78) = 6.33, p < .001, *r* = .58)

<u>*Strong Alliance Basketball Condition, (N=37)*</u>
- total errors: M = 13.7, SD = 3.79
- making more within-sex errors (M = 7.24, SD = 2.19) than between-sex errors (M = 4.84, SD = 2.27), t(36) = 4.70, p < .001, *r* = .62
- more within-team errors (M = 11.22, SD = 3.05) than between-team errors (M = 1.86, SD =2.66), t(36) = 12.12, p < .001, *r* = .90

<u>*Strong Alliance Basketball vs. Antagonistic Baseline Conditions*</u>
- decrease in categorization by sex compared to baseline (error difference in the *antagonistic baseline*: M = 5.88, SD = 4.52; error difference in the *strong basketball* condition: M = 2.40, SD = 3.11; change in categorization: t(75) = -3.91, p < .001, *r* = .41)
- substantial increase in categorization by team compared to baseline shirt color categorization (error difference in the *antagonistic baseline:* -.55, SD = 3.92; error difference in the *strong basketball* condition: M = 9.35, SD = 4.69; change in categorization: t(75) = 10.08, p < .001, *r* = .76)

<u>*Strong Alliance Basketball vs. Moderate Alliance Basketball Conditions*</u>
- no decrease in categorization by sex compared to moderate (error difference in the *moderate basketball* condition: M = 3.63, SD = 3.68; error difference in the strong *basketball* condition: M = 2.40, SD = 3.11; change in categorization: t(75) = -1.58, p = .118, *r* = .18)
- increase in categorization by team compared to moderate (error difference in the *moderate basketball* condition: M = 6.39, SD = 5.72; error difference in the strong *basketball* condition: M = 9.35, SD = 4.69; change in categorization: t(75) = 2.47, p = .016, *r* = .27)

Bayesian Analysis

All analyses were run with the BEST software for R[29] and used the noncommittal, broad default prior. The most relevant estimates for the reader are likely to be the *Difference of Means* and *Effect Size,* which can be found at the middle and bottom of the right-hand column of each figure, respectively.

<u>*Positive Baseline Condition, Targets Differing in Race*</u>

- Racial Categorization (within-subjects comparison of within-race errors versus between-race errors; Group 1 = within-race errors, Group 2 = between-race errors)

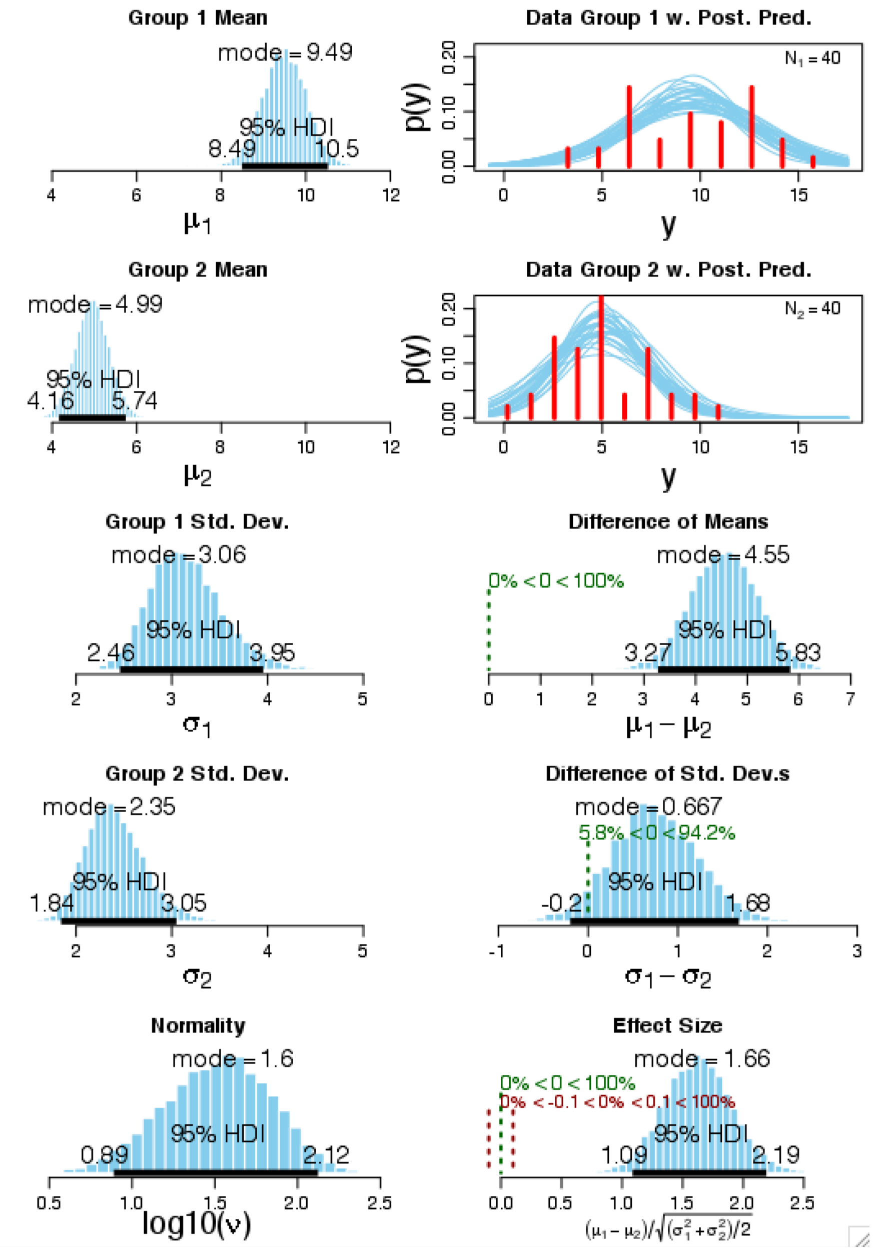

Positive Baseline Condition, Targets Differing in Race

- Shirt Color Categorization (within-subjects comparison of within-shirt-color errors versus between-shirt-color errors; Group 1 = within-shirt-color errors, Group 2 = between-shirt-color errors)

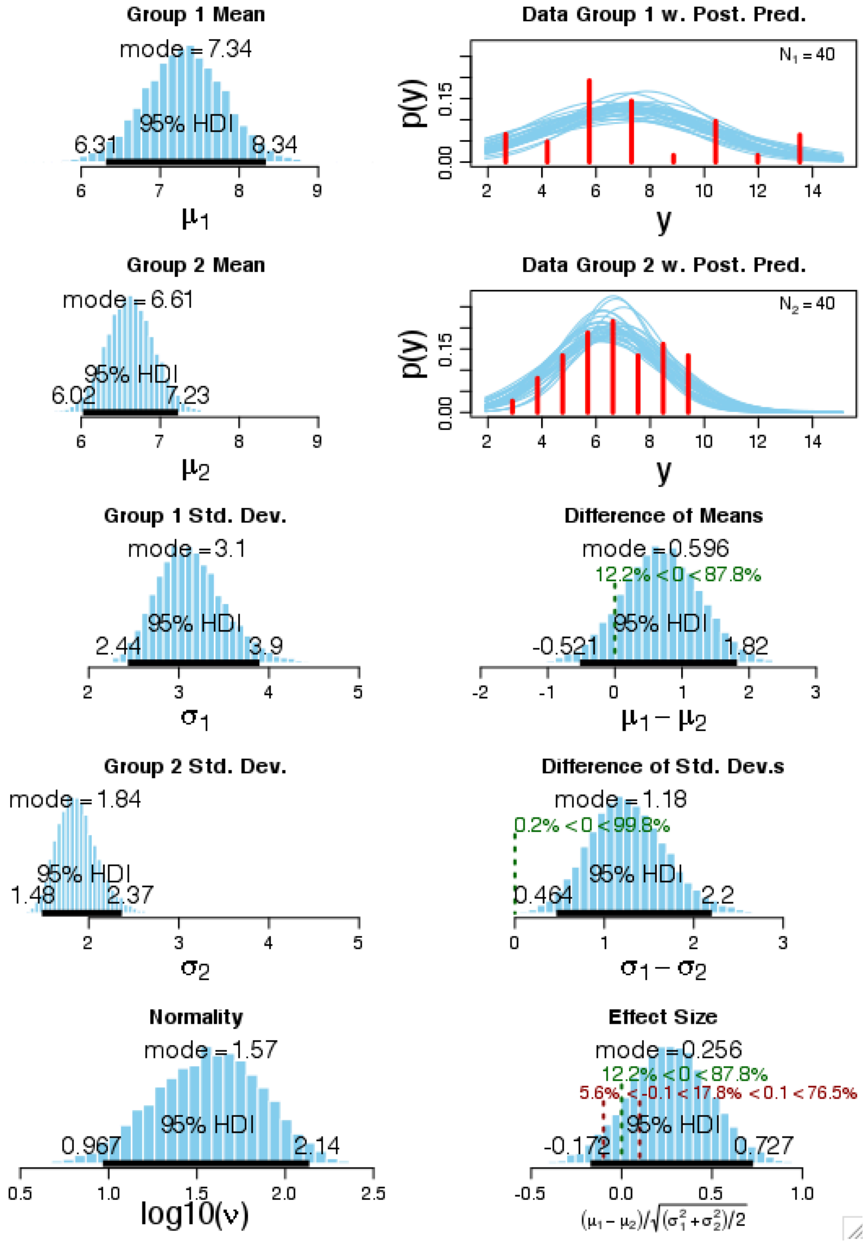

Antagonistic Baseline Condition, Targets Differing in Race

- Racial Categorization (within-subjects comparison of within-race errors versus between-race errors; Group 1 = within-race errors, Group 2 = between-race errors)

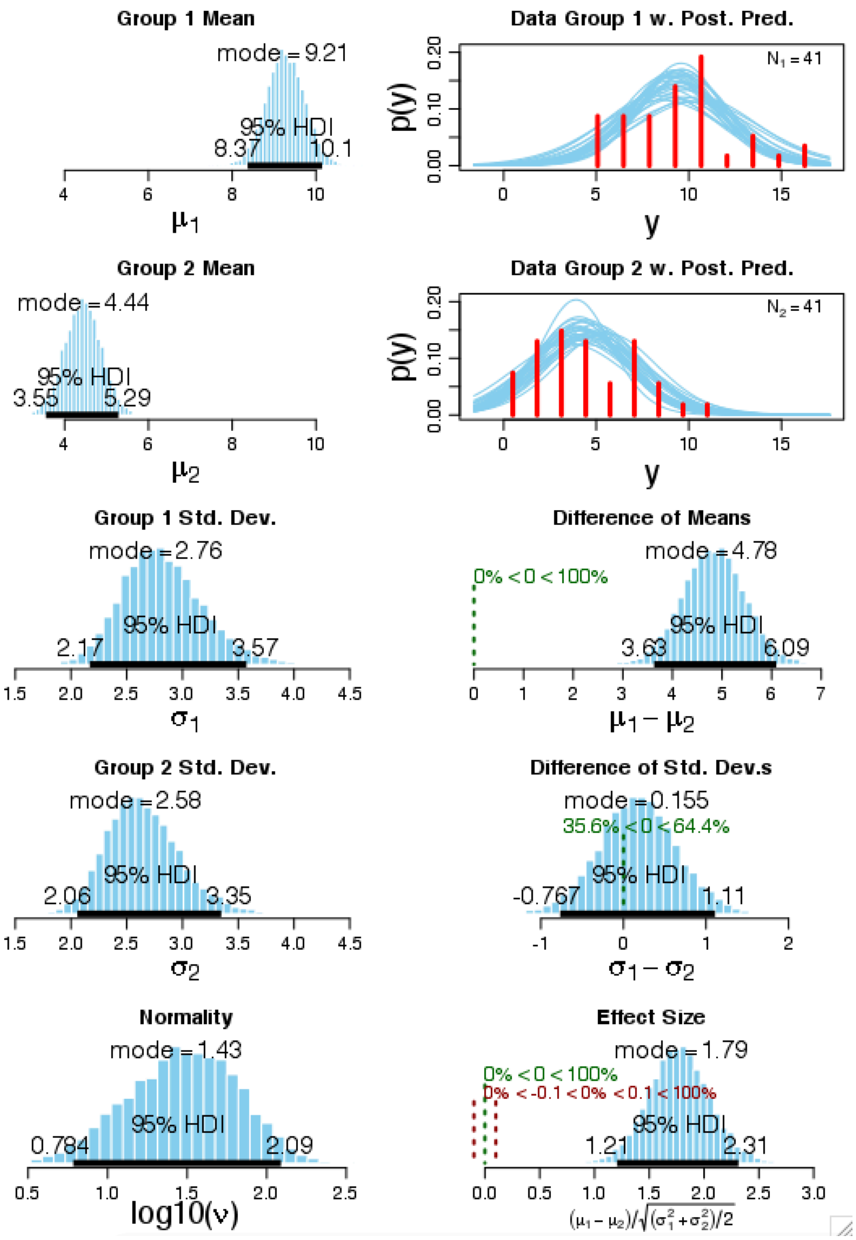

Antagonistic Baseline Condition, Targets Differing in Race

- Shirt Color Categorization (within-subjects comparison of within-shirt-color errors versus between-shirt-color errors; Group 1 = within-shirt-color errors, Group 2 = between-shirt-color errors)

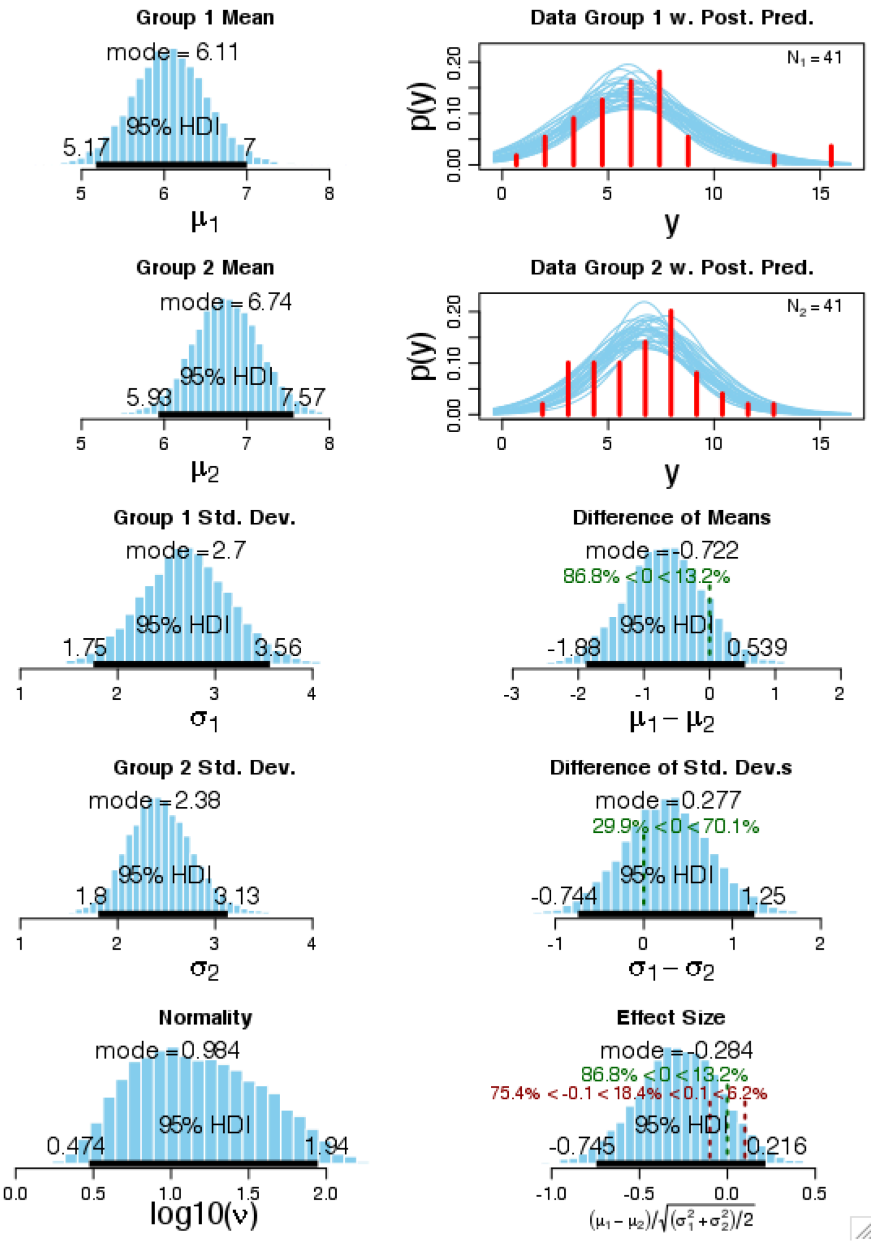

Moderate Alliance Strength Soccer Condition, Targets Differing in Race

- Racial Categorization (within-subjects comparison of within-race errors versus between-race errors; Group 1 = within-race errors, Group 2 = between-race errors)

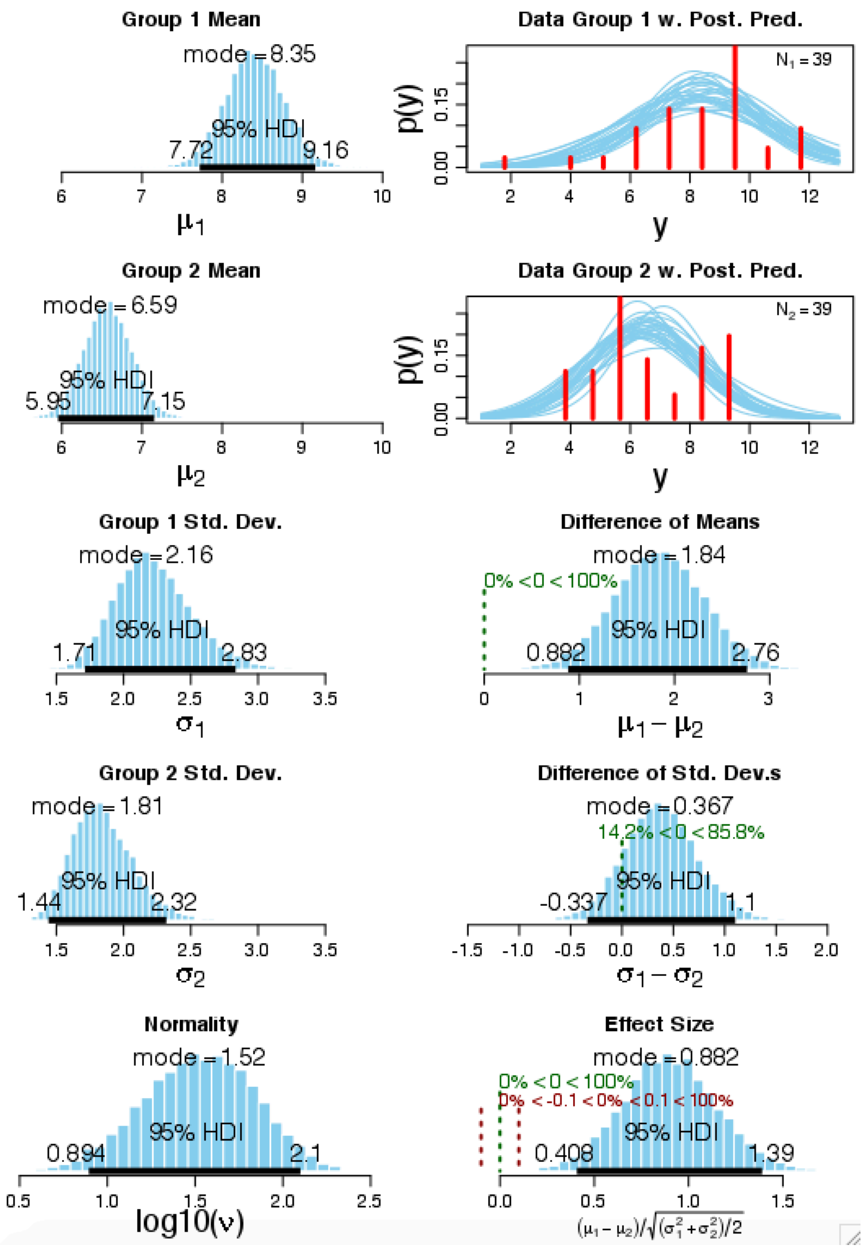

Moderate Alliance Strength Soccer Condition, Targets Differing in Race

- Team Categorization (within-subjects comparison of within-team errors versus between-team errors; Group 1 = within-team errors, Group 2 = between-team errors)

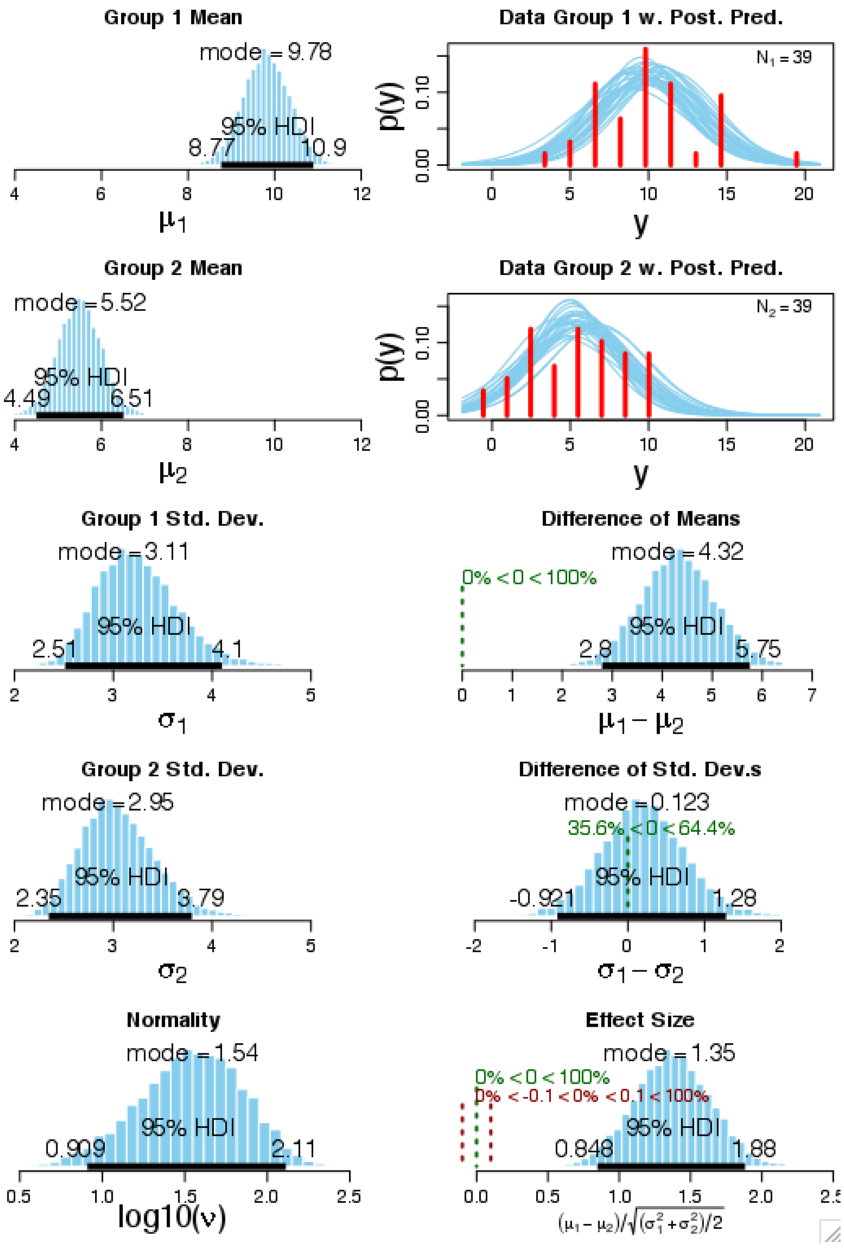

Moderate Alliance Strength Basketball Condition, Targets Differing in Race

- Racial Categorization (within-subjects comparison of within-race errors versus between-race errors; Group 1 = within-race errors, Group 2 = between-race errors)

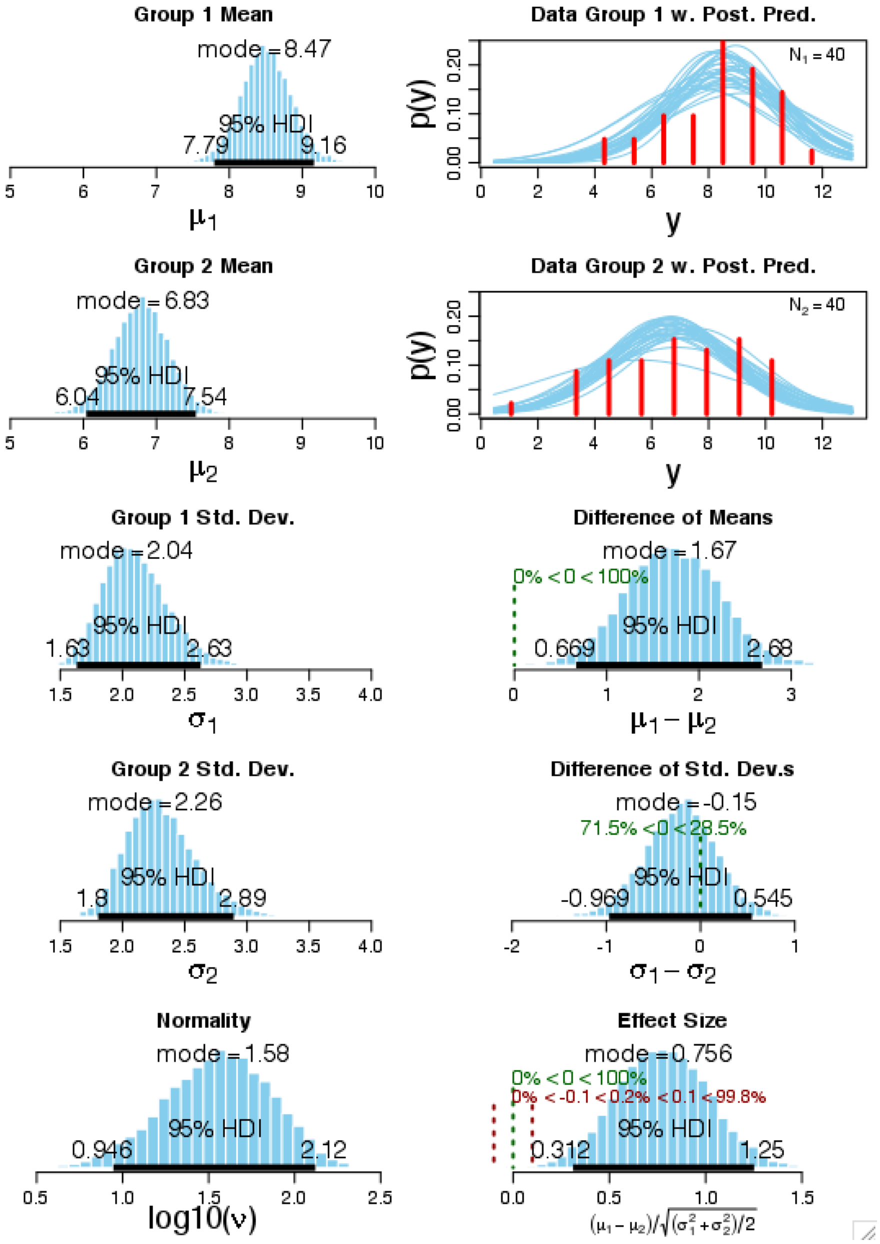

<u>*Moderate Alliance Strength Basketball Condition, Targets Differing in Race*</u>

- Team Categorization (within-subjects comparison of within-team errors versus between-team errors; Group 1 = within-team errors, Group 2 = between-team errors)

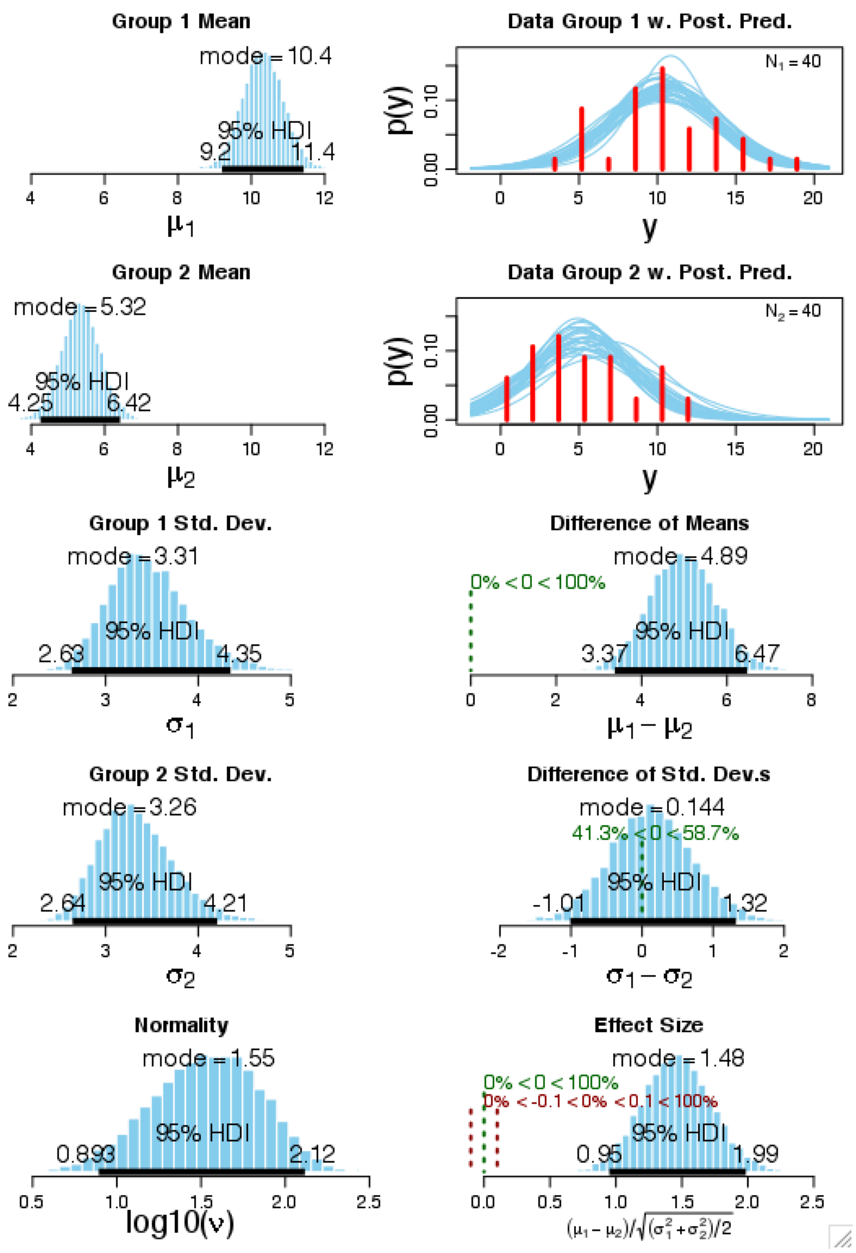

Strong Alliance Basketball Condition, Targets Differing in Race

- Racial Categorization (within-subjects comparison of within-race errors versus between-race errors; Group 1 = within-race errors, Group 2 = between-race errors)

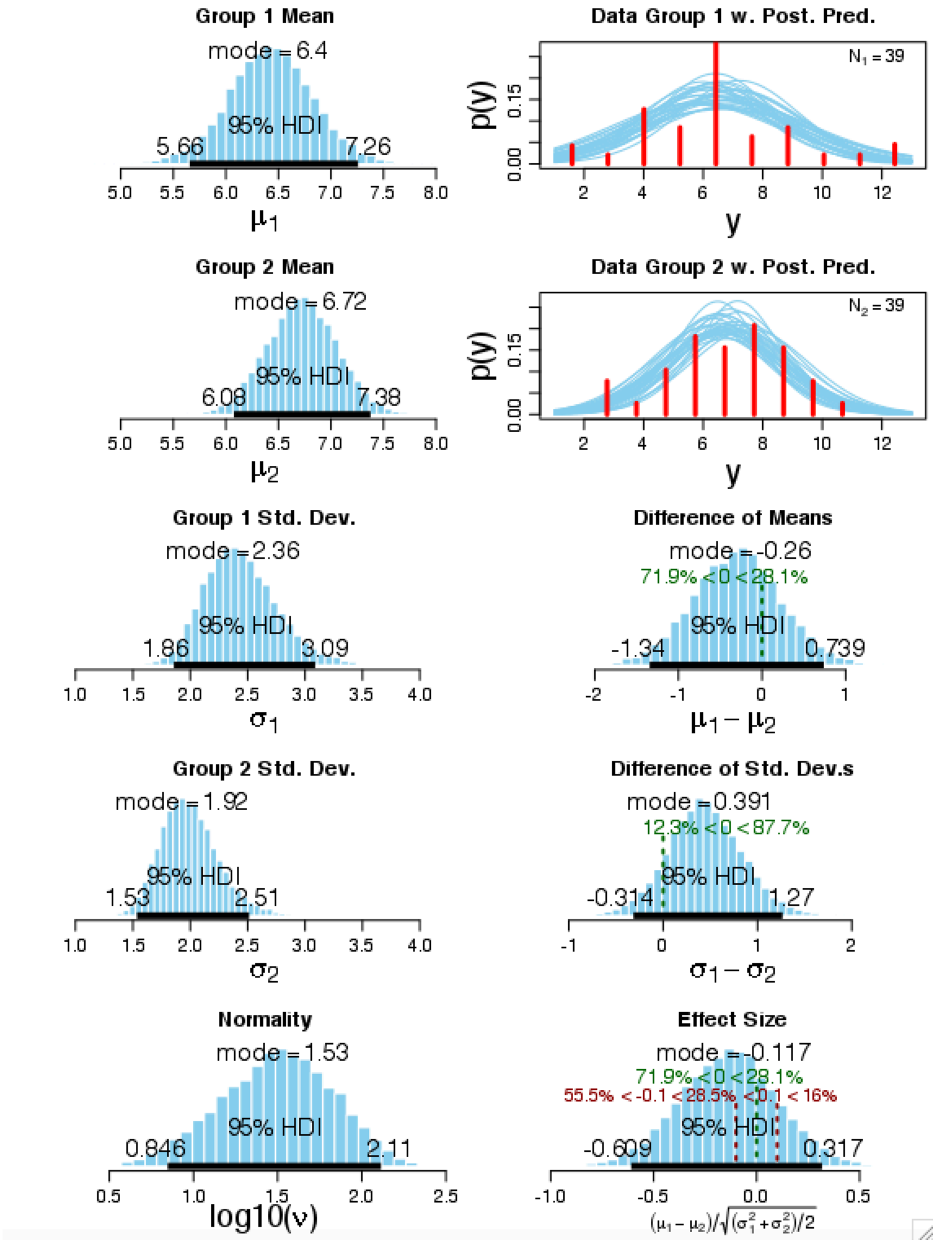

<u>Strong Alliance Basketball Condition, Targets Differing in Race</u>

- Team Categorization (within-subjects comparison of within-team errors versus between-team errors; Group 1 = within-team errors, Group 2 = between-team errors)

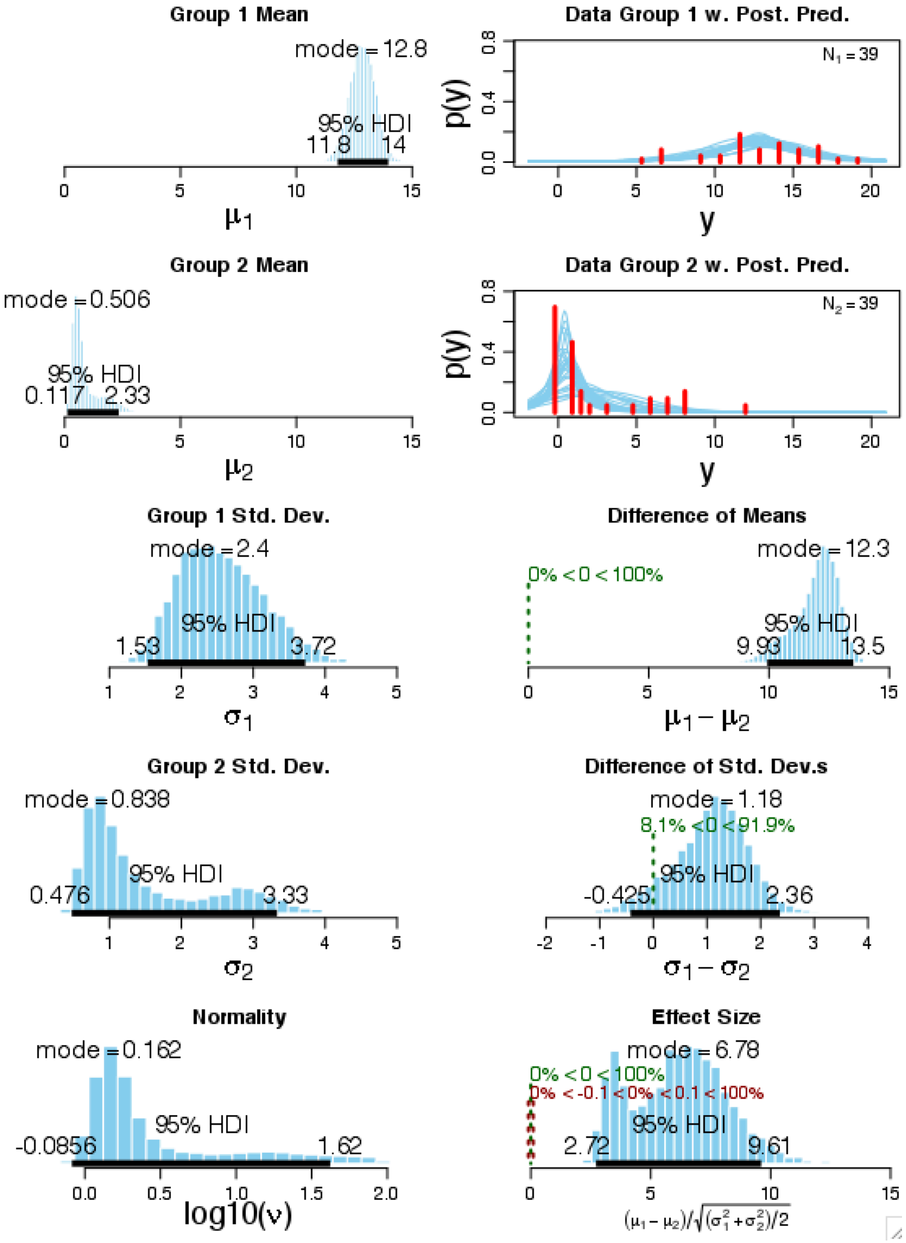

Positive Baseline Condition, Targets Differing in Sex

- Sex Categorization (within-subjects comparison of within-sex errors versus between-sex errors; Group 1 = within-sex errors, Group 2 = between-sex errors)

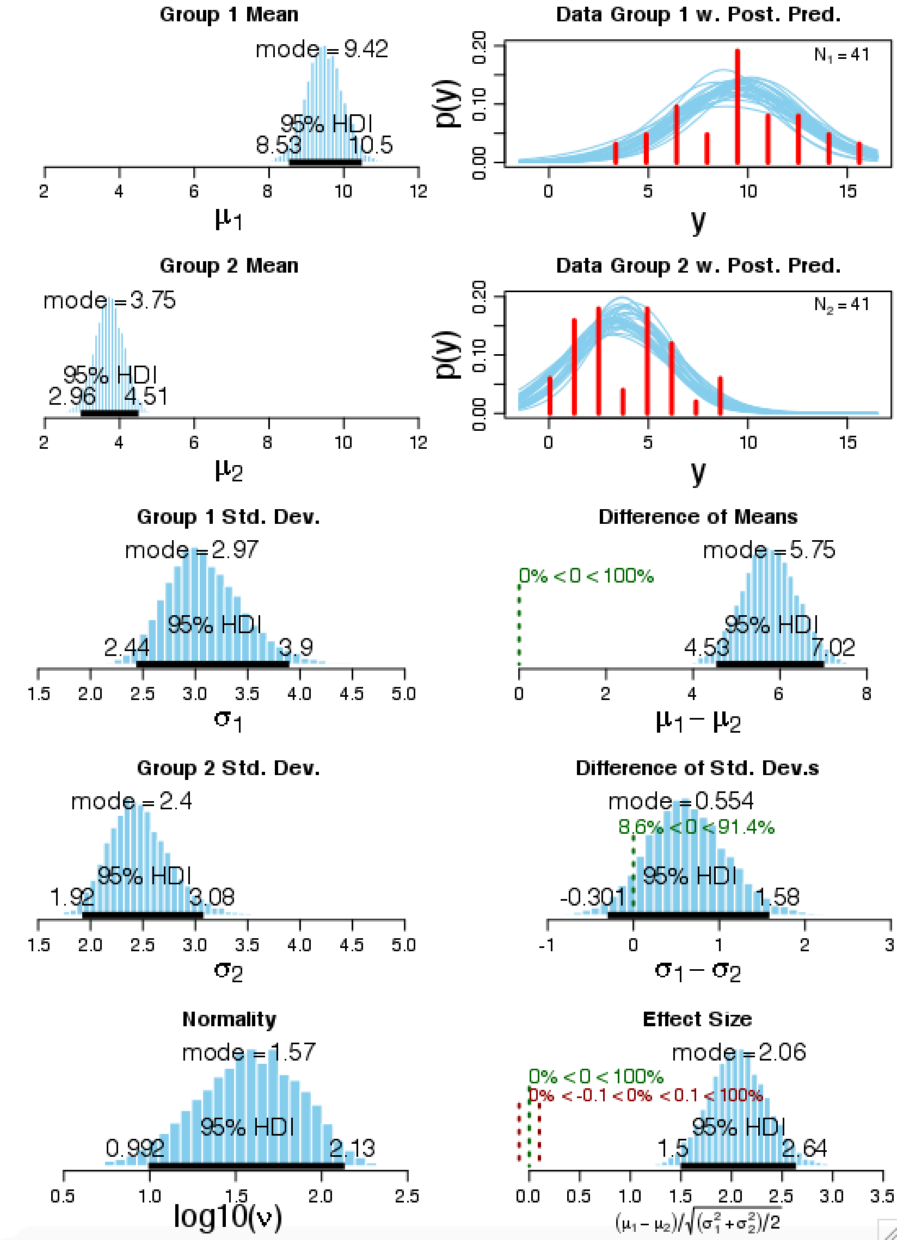

Positive Baseline Condition, Targets Differing in Sex

- Shirt Color Categorization (within-subjects comparison of within-shirt-color errors versus between-shirt-color errors; Group 1 = within-shirt-color errors, Group 2 = between-shirt-color errors)

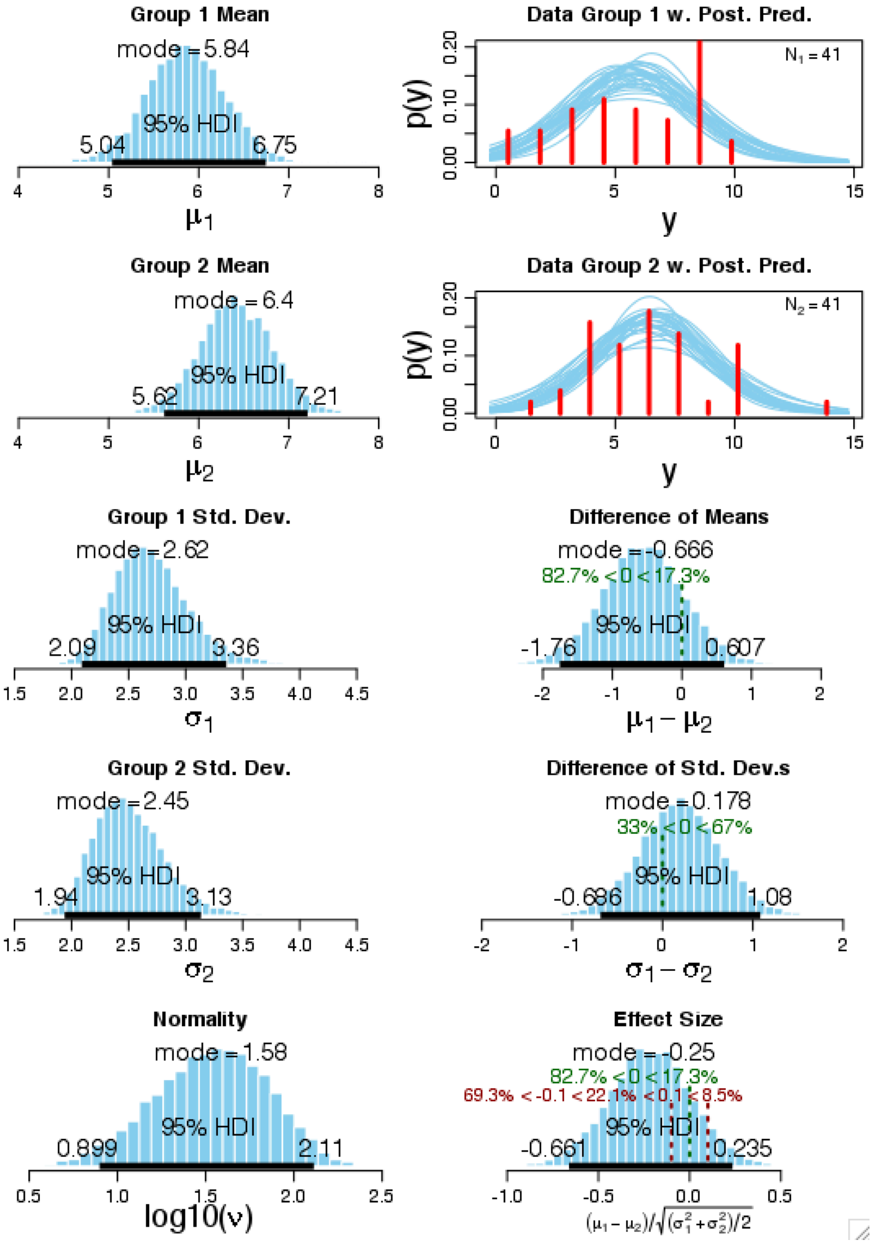

Antagonistic Baseline Condition, Targets Differing in Sex

- Sex Categorization (within-subjects comparison of within-sex errors versus between-sex errors; Group 1 = within-sex errors, Group 2 = between-sex errors)

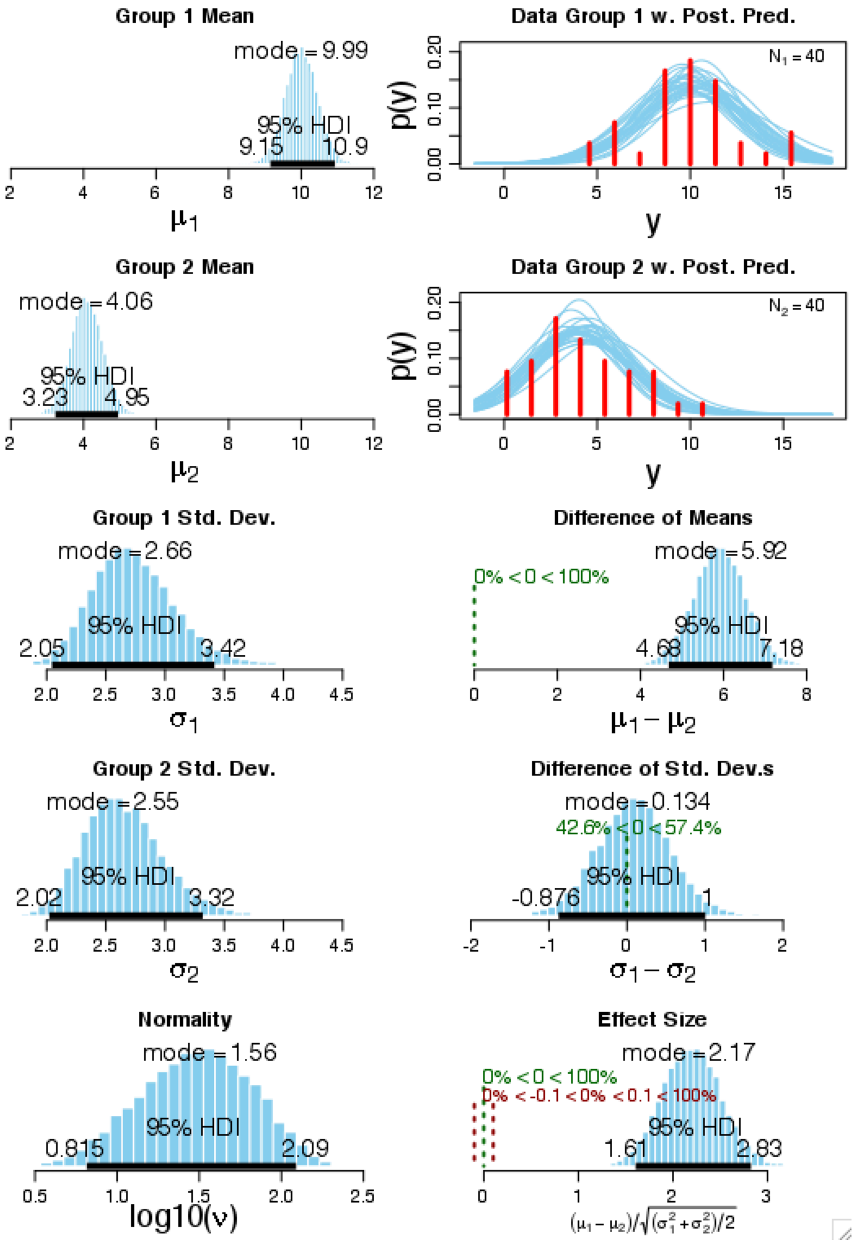

<u>Antagonistic Baseline Condition, Targets Differing in Sex</u>

- Shirt Color Categorization (within-subjects comparison of within-shirt-color errors versus between-shirt-color errors; Group 1 = within-shirt-color errors, Group 2 = between-shirt-color errors)

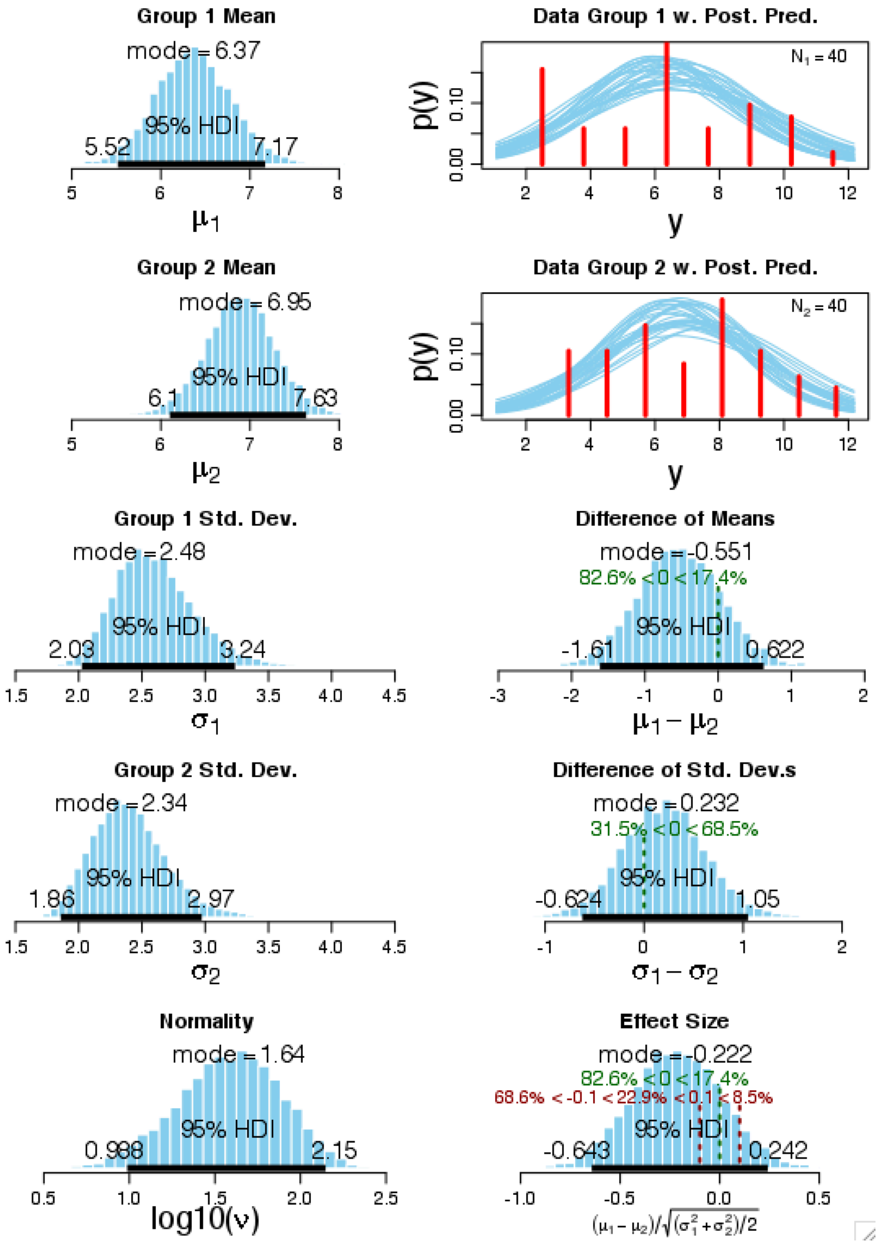

Moderate Alliance Strength Soccer Condition, Targets Differing in Sex

- Sex Categorization (within-subjects comparison of within-sex errors versus between-sex errors; Group 1 = sex-race errors, Group 2 = between-sex errors)

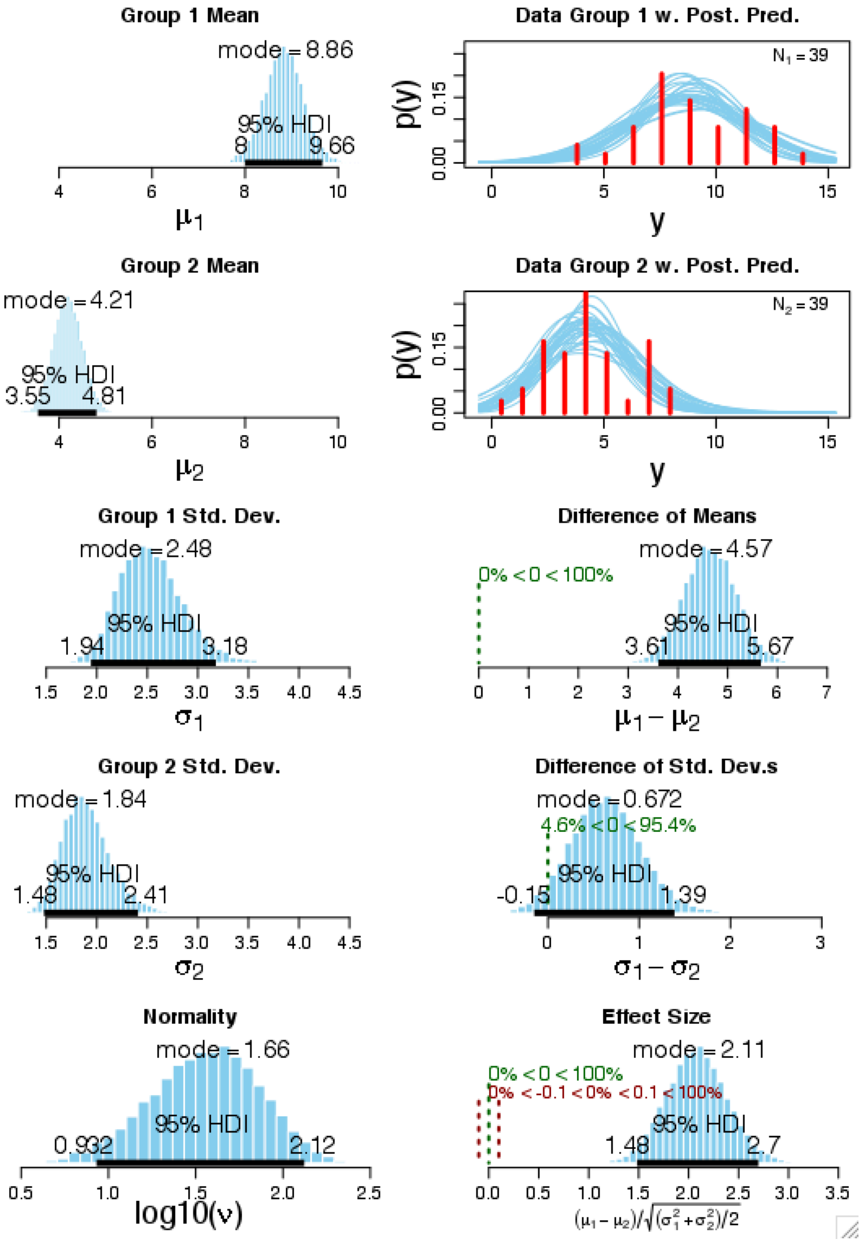

Moderate Alliance Strength Soccer Condition, Targets Differing in Sex

- Team Categorization (within-subjects comparison of within-team errors versus between-team errors; Group 1 = within-team errors, Group 2 = between-team errors)

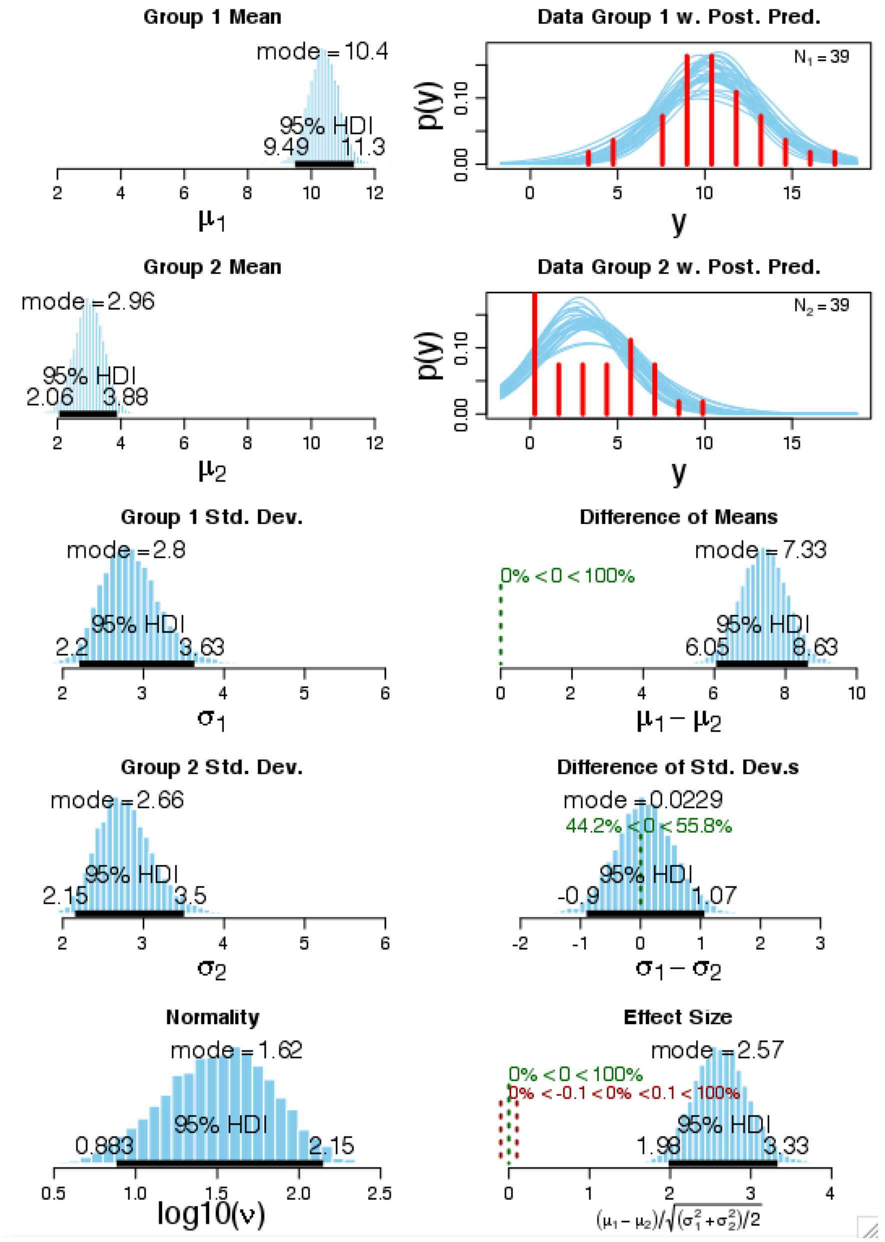

Moderate Alliance Strength Basketball Condition, Targets Differing in Sex

- Sex Categorization (within-subjects comparison of within-sex errors versus between-sex errors; Group 1 = within-sex errors, Group 2 = between-sex errors)

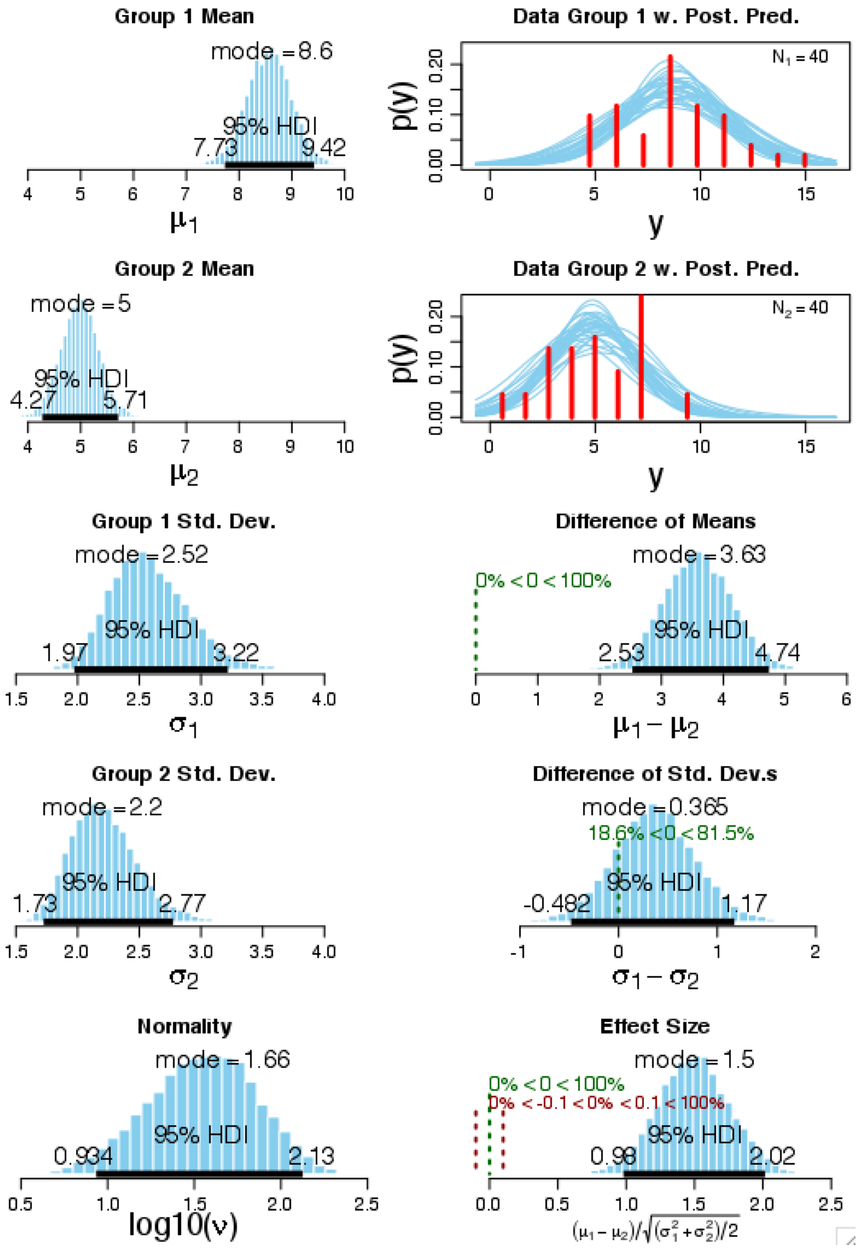

Moderate Alliance Strength Basketball Condition, Targets Differing in Sex

- Team Categorization (within-subjects comparison of within-team errors versus between-team errors; Group 1 = within-team errors, Group 2 = between-team errors)

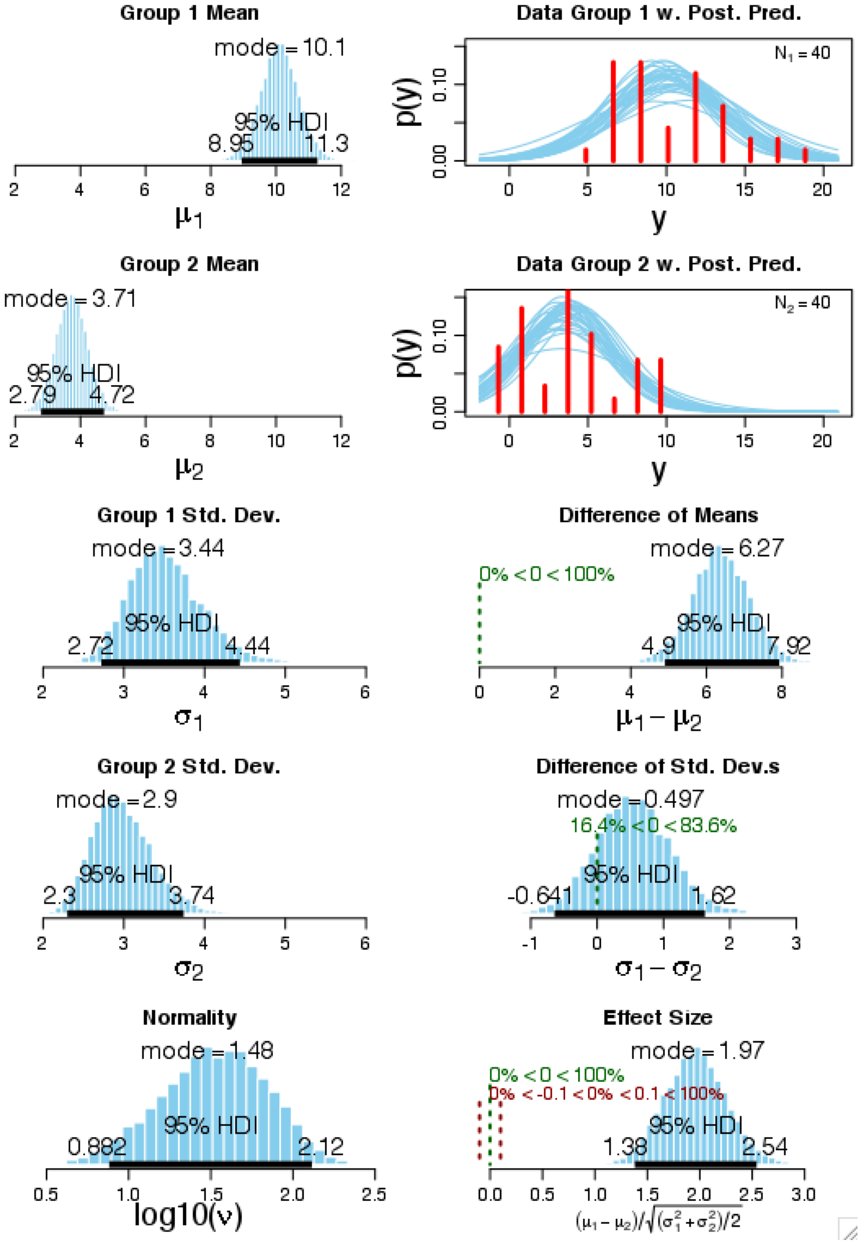

<u>*Strong Alliance Basketball Condition, Targets Differing in Sex*</u>

- Sex Categorization (within-subjects comparison of within-sex errors versus between-sex errors; Group 1 = within-sex errors, Group 2 = between-sex errors)

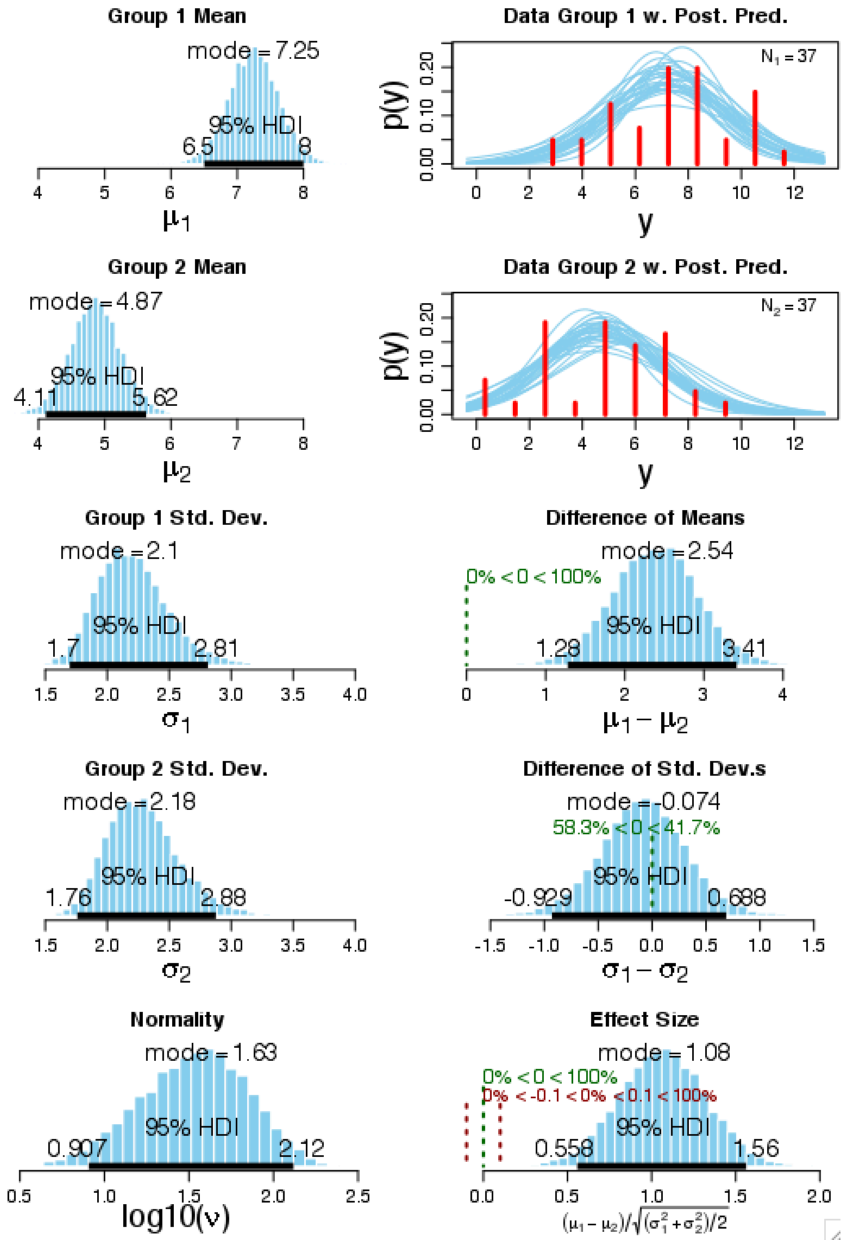

<u>*Strong Alliance Basketball Condition, Targets Differing in Sex*</u>

- Team Categorization (within-subjects comparison of within-team errors versus between-team errors; Group 1 = within-team errors, Group 2 = between-team errors)

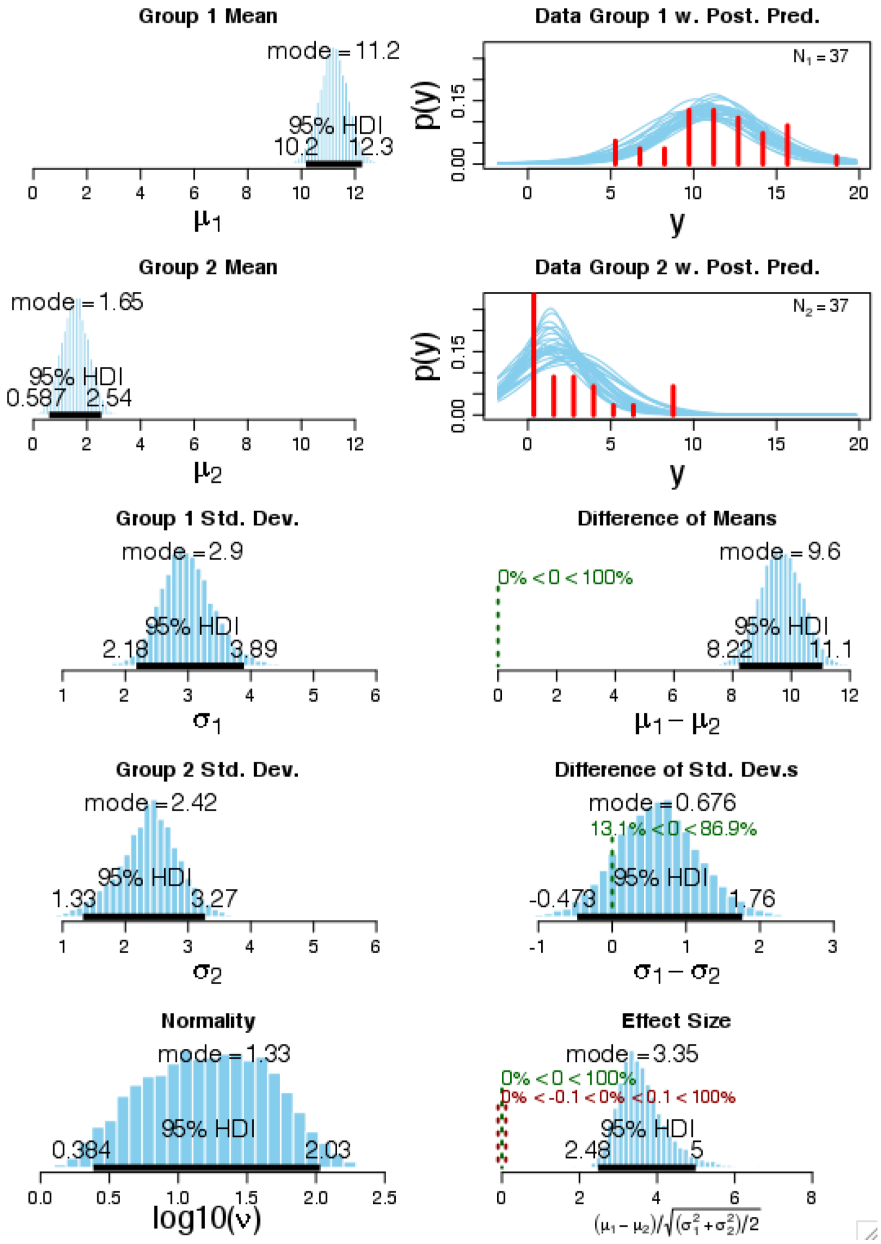

Antagonistic Baseline Condition vs. Moderate Soccer Condition, Targets Differing in Race

- Change in Race Categorization (between-subjects comparison of race error difference across the two conditions; Group 1 = Antagonistic Baseline Condition, Group 2 = Soccer Condition)

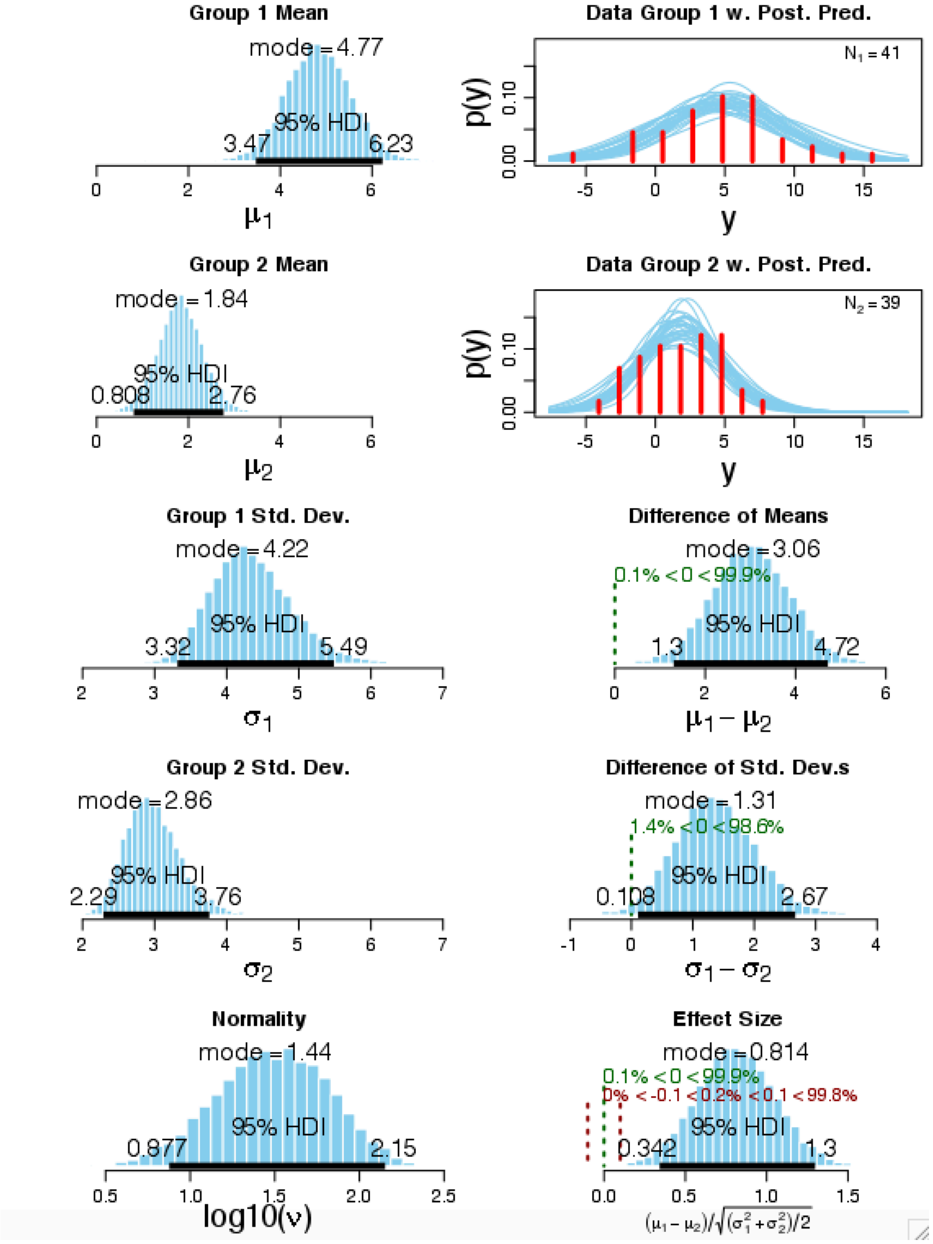

<u>*Antagonistic Baseline Condition vs. Moderate Basketball Condition, Targets Differing in Race*</u>

- Change in Race Categorization (between-subjects comparison of race error difference across the two conditions; Group 1 = Antagonistic Baseline Condition, Group 2 = Basketball Condition)

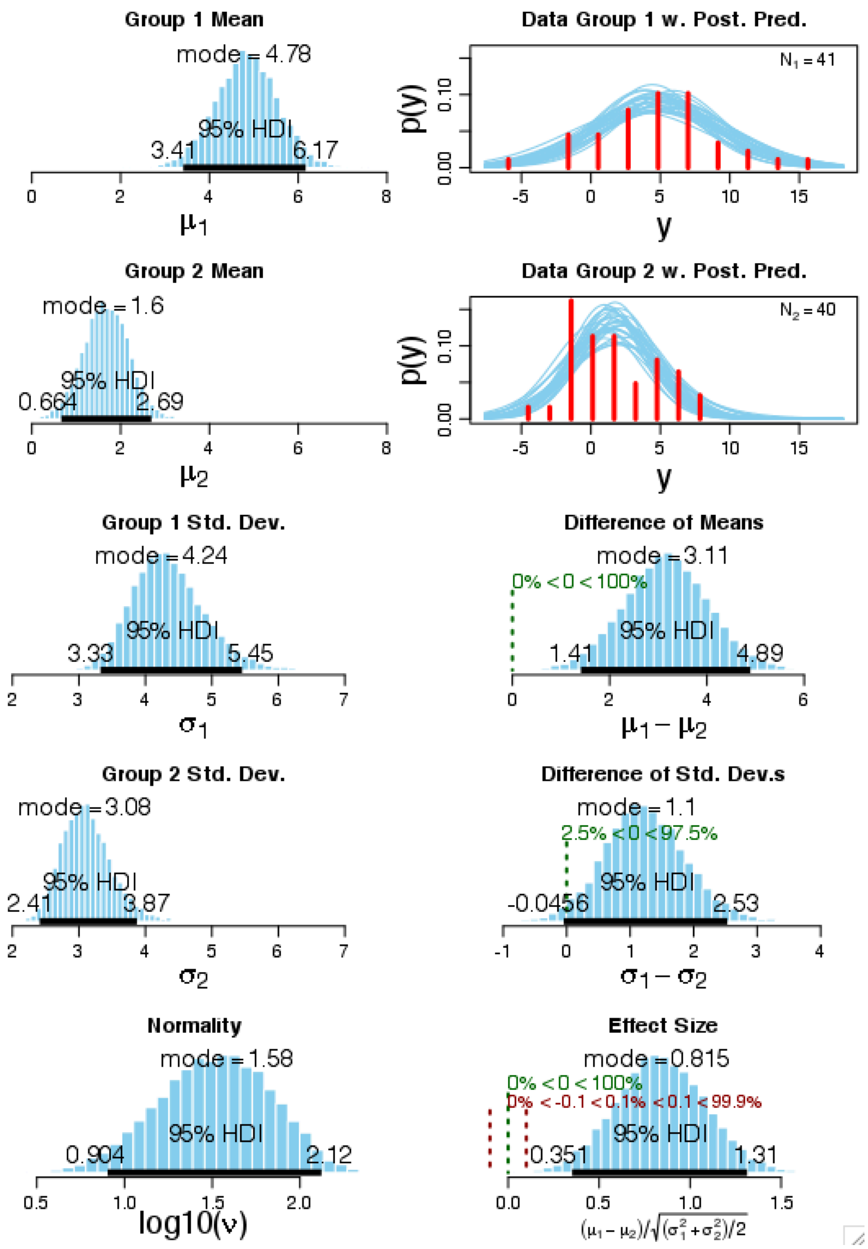

Antagonistic Baseline Condition vs. Strong Basketball Condition, Targets Differing in Race

- Change in Race Categorization (between-subjects comparison of race error difference across the two conditions; Group 1 = Antagonistic Baseline Condition, Group 2 = Basketball Condition (with Artifact))

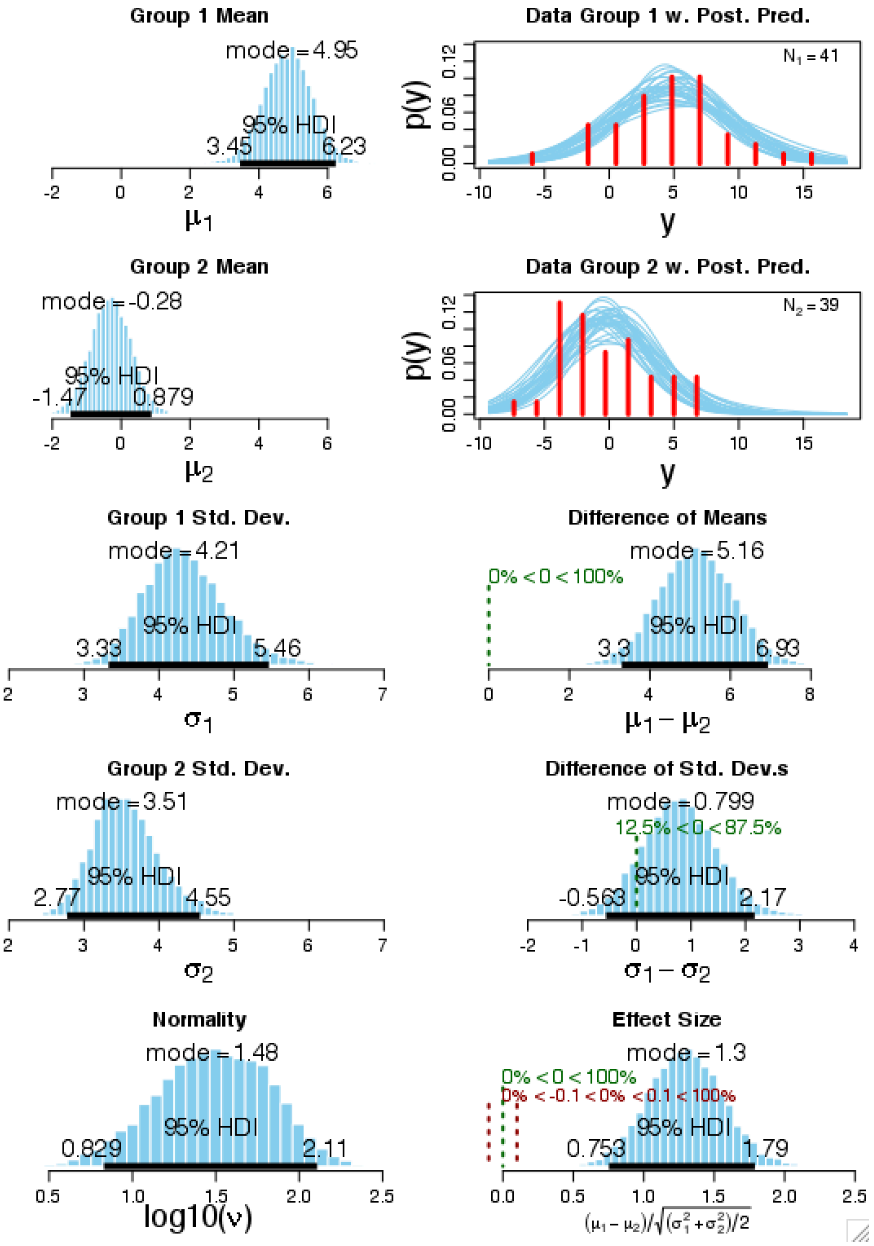

Antagonistic Baseline Condition vs. Moderate Soccer Condition, Targets Differing in Sex

- Change in Sex Categorization (between-subjects comparison of sex error difference across the two conditions; Group 1 = Antagonistic Baseline Condition, Group 2 = Soccer Condition)

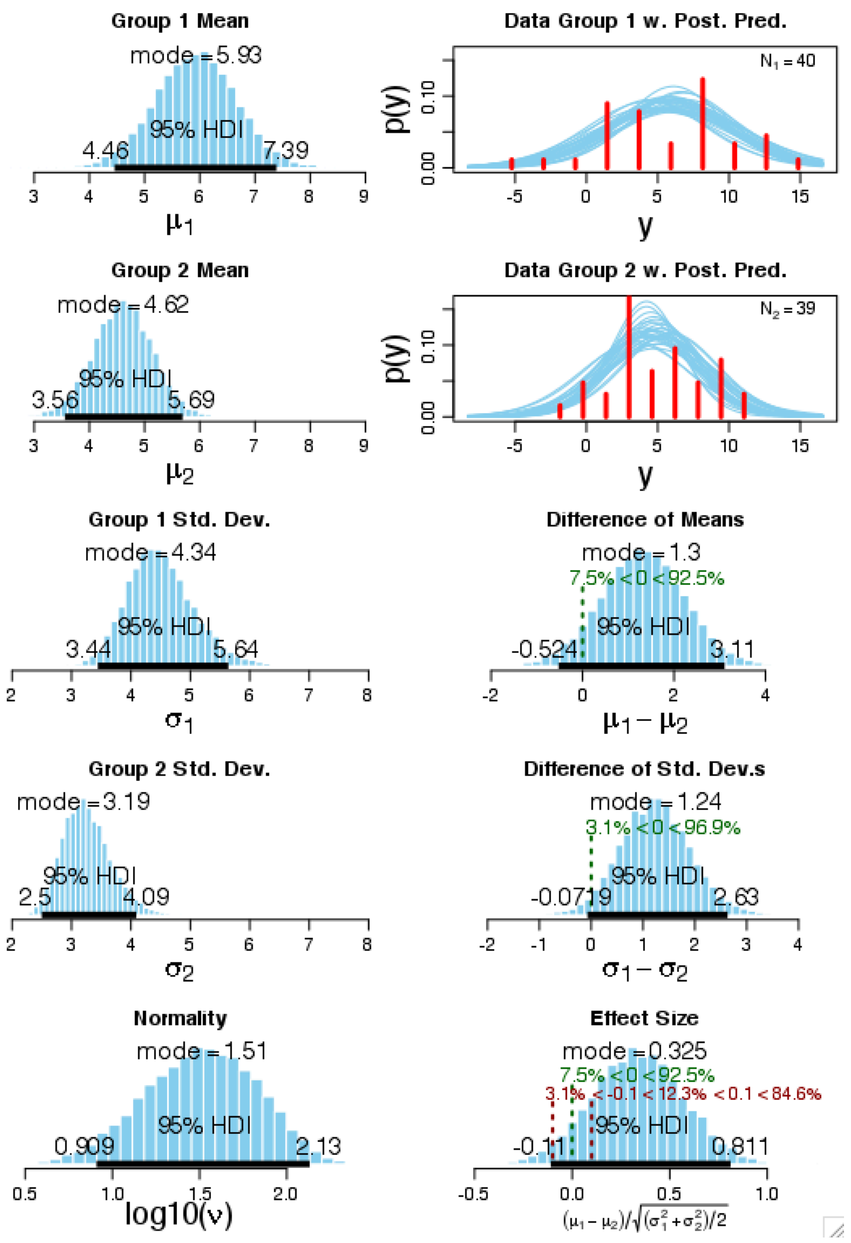

<u>*Antagonistic Baseline Condition vs. Moderate Basketball Condition, Targets Differing in Sex*</u>

- Change in Sex Categorization (between-subjects comparison of sex error difference across the two conditions; Group 1 = Antagonistic Baseline Condition, Group 2 = Basketball Condition)

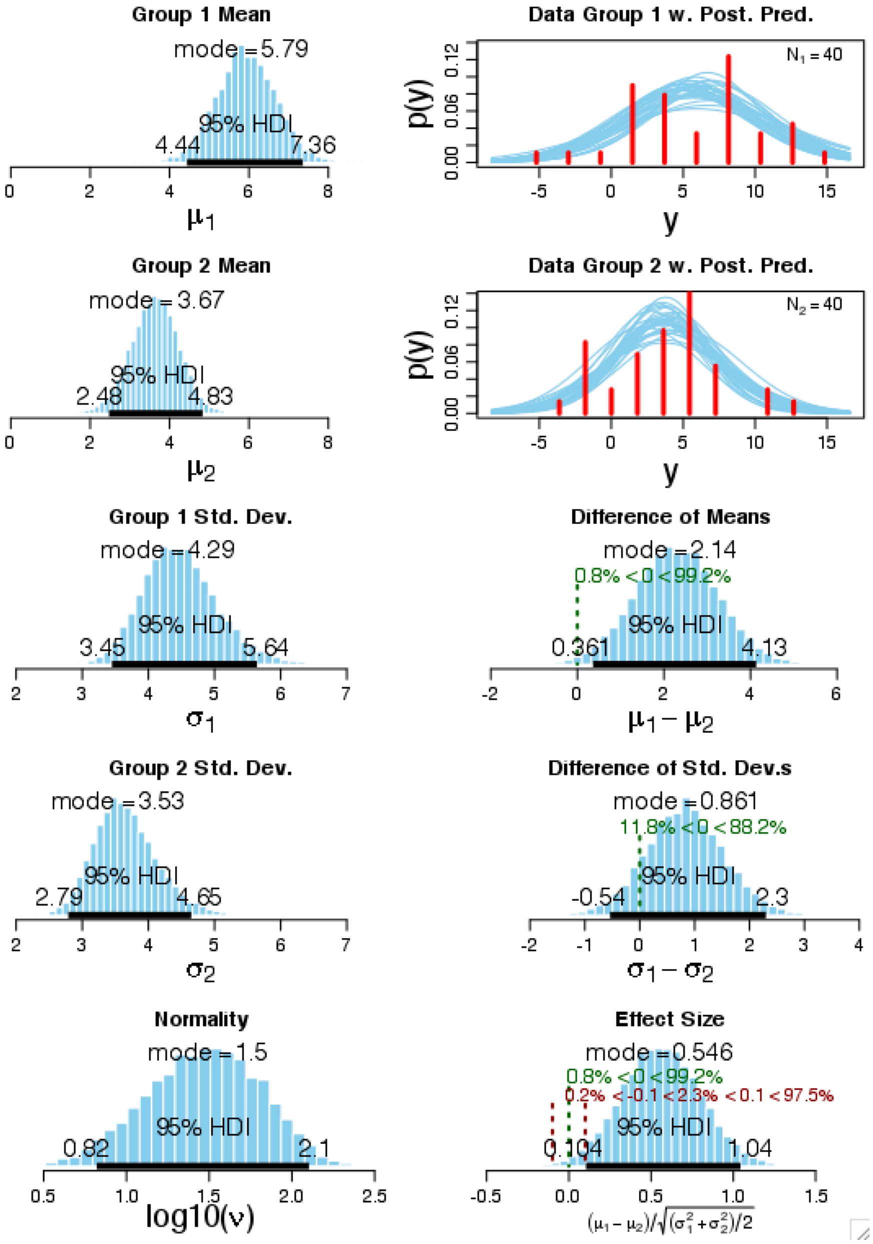

<u>*Antagonistic Baseline Condition vs. Strong Basketball Condition, Targets Differing in Sex*</u>

- Change in Sex Categorization (between-subjects comparison of sex error difference across the two conditions; Group 1 = Antagonistic Baseline Condition, Group 2 = Basketball Condition(with Artifact))

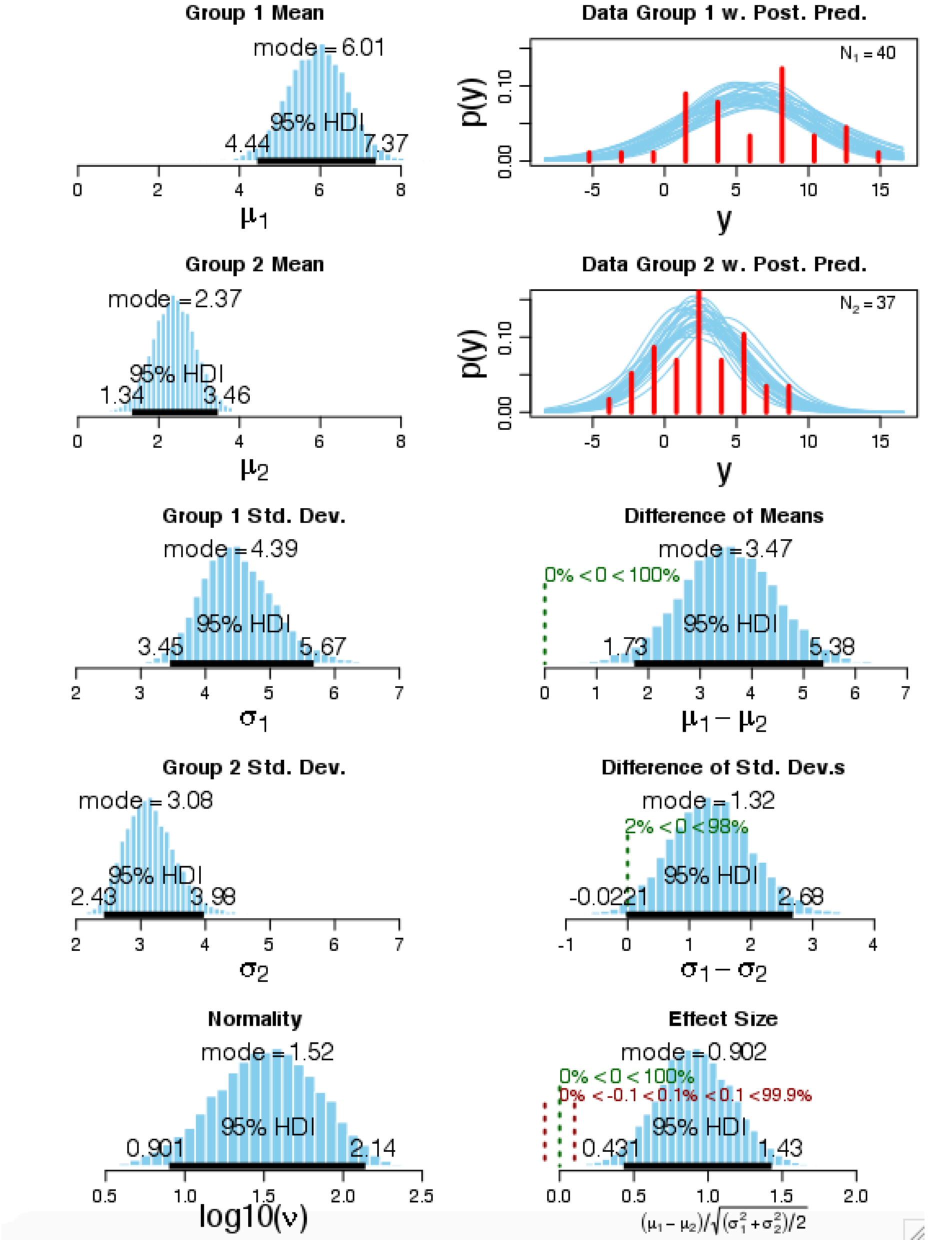

Supplement: Supplementary file 2 — Supplementary Information 2. [file 41598_2021_82975_MOESM2_ESM.pdf]
